# Mitochondrial Dysfunction Contributes to Sustained Muscle Loss After Cardiac Surgery: A Prospective Observational Study

**DOI:** 10.1002/jcsm.70051

**Published:** 2025-08-21

**Authors:** Ashley N. Thomas, Antonis Kalakoutas, Martin Yates, John Yap, Julie Sanders, Paul Kemp, Mark J. D. Griffiths

**Affiliations:** ^1^ Barts Heart Centre St Bartholomew's Hospital, Barts NHS Trust London UK; ^2^ Guy's and St Thomas' NHS Trust London UK; ^3^ Florence Nightingale Faculty of Nursing, Midwifery and Palliative Care Kings College London UK; ^4^ National Heart and Lung Institute Imperial College London UK

**Keywords:** aortic surgical procedures, critical illness myopathy, metabolomics, mitochondria, muscle regeneration, muscle strength recovery, muscular atrophy, pathology

## Abstract

**Background:**

As a major systemic insult, cardiac surgery can lead to significant muscle loss, which increases the time to recovery as well as being correlated with mortality. Highly variable loss of muscle mass (0%–40% rectus femoris cross‐sectional area [RFcsa]) and strength in the week after surgery has aided understanding of mechanisms of sarcopenia after acute illness. To include muscle recovery, patients' muscle phenotype beyond the first week after surgery and up to their return as outpatients was studied and correlated with protein and metabolomic markers.

**Methods:**

Patients undergoing elective aortic valve surgery were recruited. Muscle mass (RFcsa), strength (handgrip, knee extension and spirometry), body composition (by bioimpedance) and health‐related quality of life (generic questionnaire EQ‐5D‐5L) were determined pre‐operatively, 7 days after surgery and at outpatient follow‐up. Blood samples were taken on Days 0, 1, 3, 7 and follow‐up. The plasma metabolome was determined in 20 patients at Days 0, 3, 7 and follow‐up.

**Results:**

Of 31 participants, 20 were male: mean age 68.8 years with a range between 48 and 85 years. Proportionate mean loss of RFcsa between pre‐op and Day 7 values was 6.44% [95% CI 4.21 to 8.68, *n* = 31]; between pre‐op and follow‐up 9.69% [95% CI 4.92 to 14.96, *n* = 22]; and between Day 7 and follow‐up 3.60% [95% CI −1.30 to 8.48, *n* = 22]. By contrast to measures of muscle bulk, the strength and functionality assessments (knee extension, handgrip, spirometry and short physical performance battery) decreased in the first week after surgery (pre‐op to Day 7) followed by a return to baseline (Day 7 to follow‐up). Health‐related quality of life (cross‐walk index) changed little over the course of the study but correlated positively at follow‐up with muscle bulk (RFcsa: *r* = 0.58 [95% CI 0.19 to 0.81] *p* = 0.005) and strength of knee extension (*r* = 0.54 [95% CI 0.14 to 0.79] *p* = 0.010) and handgrip (*r* = 0.63 [95% CI 0.27 to 0.83] *p* = 0.002: *n* = 22). Both pre‐operative and peak (Day 3) plasma levels of short‐chain acyl‐carnitine markers of mitochondrial dysfunction correlated with proportional muscle loss at follow‐up and with strength at all timepoints.

**Conclusions:**

Prolonged follow‐up after aortic surgery demonstrated a divergence between the consistent recovery of strength and a significant proportion of patients continuing to lose muscle bulk. Markers of baseline and acute mitochondrial dysfunction predicted poor muscle outcomes up to outpatient follow‐up.

## 1 Introduction

Rapid and sometimes severe muscle wasting and weakness are common features of critical illness. This phenomenon, termed ICU‐acquired weakness (ICUAW), increases the time required to wean patients from assisted ventilation, which dramatically prolongs the time spent in ICU and thereby contributes to mortality [1]. ICUAW also accounts for considerable long‐term disability with important functional and fiscal consequences. For example, approximately half of patients surviving the acute respiratory distress syndrome (ARDS) were unable to return to work 18 months after hospital discharge. Furthermore, ARDS survivors performed significantly below par in tests of physical function 5 years after critical illness [2]. There are no disease‐modifying treatments for ICUAW.

Our recent work has characterised muscle signalling pathways that mediate muscle breakdown after cardiac surgery, effectively a human model of ICUAW [3, 4]. Specifically, we found that 50% of patients lost significant (≥ 10%) muscle mass in the 7 days after uncomplicated surgery. Patients lost between 0% and 40% of the cross‐sectional area of the rectus femoris (RFcsa) over this period, and the extent of muscle loss was not predicted by standard measures of disease severity or intra‐operative factors [3]. Hence, as in a variety of acute and chronic conditions, disease severity and muscle wasting correlate poorly. This is probably a result of genetic and epigenetic factors that contribute to predisease muscle mass and individual's susceptibility to the stress imposed by disease [5]. Understanding individual susceptibility to disease‐associated muscle wasting is important for two reasons: firstly, because of the well‐documented effects of reduced muscle mass and strength on health outcomes, and secondly, in trials of anabolic agents, the variability of response increases markedly the number of subjects required in clinical trials.

The mechanisms underlying ICUAW are incompletely understood [6]. However, the loss of muscle occurs most rapidly in the initial period of critical illness in response to inflammation and oxidative stress owing to a decreased rate of muscle protein synthesis and an increased rate of breakdown with a net negative protein balance. The loss of muscle mass can occur despite adequate nutrition and cannot be explained by immobility alone. Comparing circulating metabolites using an unbiased approach in patients who wasted significantly in the first week after aortic surgery with those who did not, we identified pre‐existing mitochondrial dysfunction as a factor associated with muscle loss [7]. Mitochondrial dysfunction, a consequence of the activation of multiple catabolic pathways [8], is a proposed common factor in muscle wasting from a variety of causes, including increased expression of miR‐542 in similar patients [9].

The determinants and mediators of muscle recovery after critical illness have been relatively overlooked. However, the recovery phase is particularly important because ICUAW is initially hidden in unconscious patients, so that once weakness becomes apparent on waking, the condition is established. Hence, we performed a further observational study in patients undergoing elective aortic valve surgery with an extended period of follow‐up to characterise longer‐term muscle dysfunction and its effects on health‐related quality of life. At the same time, we analysed patient plasma in the peri‐operative period for circulating metabolites, as well as known and proposed mediators of muscle dysfunction to evaluate pathways of interest. This enabled us to explore mediators of long‐term recovery and to validate biomarkers of muscle loss from previous studies.

## 2 Methods

### 2.1 Patients

The patients eligible for inclusion in this study were over 18 years and scheduled for elective aortic surgery at St Bartholomew's Hospital. Exclusion criteria included neuromuscular conditions (most likely stroke) affecting muscle strength that predated or complicated surgery and a prolonged stay in ICU (≥ 7 days).

### 2.2 Muscle Assessments

Muscle bulk as RFcsa was measured by a single trained practitioner in both legs under, as far as possible, identical conditions within a week of surgery pre‐op (Day 0), on Day 7 post‐operatively or at hospital discharge if sooner (Day 7) and at outpatient follow‐up in both legs by ultrasound (Fujifilm, SonoSite M‐Turbo, SonoSite Ltd., Bedford, UK) as previously described [10]. As previously, patients were categorised into ‘wasters’ and ‘non‐wasters’ depending on whether the RFcsa decreased by ≥ 10% (based on the variance of the ultrasound measurement during tests of intra‐operator reproducibility for multiple users, a significant difference of approximately 7%–8% was statistically significant) between given timepoints [4, 7, 9, 11]. As an index of muscle quality, the pixel index was calculated from the RFcsa image using Photoshop (Adobe Inc. 2019 https://www.adobe.com/products/photoshop.html) as previously described [12]. Body composition by bioelectrical impedance (Bodystat 1500 device, Isle of Man, UK) and muscle strength and function were quantified at the same time as the ultrasounds by handgrip (Jamar hydraulic hand dynamometer, JA Preston Corporation, Clifton, NJ., USA), knee dynamometry (Lafayette Manual Muscle Tester, Leicestershire, UK), lying and standing spirometry (Micro 1, CareFusion Corporation, Basingstoke, UK) and the short physical performance battery (SPPB) [13].

### 2.3 Blood Samples and Analysis

Blood samples were taken into EDTA tubes before the induction of anaesthesia on the day of surgery (Day 0) and on Days 1, 3 and 7 after surgery and at the first outpatient follow‐up appointment. Plasma was prepared and stored at −80°C until required. The plasma levels of 134 metabolites were determined by reverse phase liquid chromatography and mass spectroscopy (LC–MS; Table S1) before surgery (pre‐op or Day 0) and on Days 3, 7 and at follow‐up. White blood cell count and C‐reactive protein were measured pre‐ and post‐operatively as part of routine clinical care in the hospital laboratories. Growth and differentiation factor (GDF)‐15, insulin‐like growth factor (IGF)‐1 and fibroblast growth factor (FGF)‐21 were quantified separately by ELISA according to the manufacturer's instructions (R&D systems, Abingdon, UK).

### 2.4 Health‐Related Quality of Life

This was measured pre‐operatively, on Day 7 after surgery and at follow‐up using the generic questionnaire EQ‐5D‐5L (EuroQol Research Foundation, Rotterdam, The Netherlands with permission). The five constituent domains (mobility, self‐care, usual activities, pain/discomfort and anxiety/depression) were converted to a single value: the cross‐walk index. Permission to use the questionnaire was granted from the EuroQol Research Foundation, Rotterdam, Netherlands (licence numbers 23 720/23467).

### 2.5 Statistical Methods

As this was a pilot study investigating recovery in this model for the first time, it was not possible to do a power calculation; however, our target for recruitment based on our experience was estimated as 50 patients. Data from patients completing the in‐hospital initial study requirements but unable to return for follow‐up were included for the earlier timepoints only.

Results given as mean with standard deviation (SD) or median with interquartile ranges for non‐normally distributed data determined by the Kolmogorov–Smirnov test. Student's *t*‐tests (for normally distributed data) or Mann–Whitney tests were used where appropriate for between‐group analyses. Statistical analysis and figure construction were carried out using GraphPad PRISM 10 (GraphPad Software, California, USA). Differences between data sets were analysed by ANOVA using Tukey's range test: A *p*‐value of < 0.05 was considered statistically significant, unless otherwise stated. Correlations of interest were calculated using Spearman's analysis for non‐normally distributed data.

### 2.6 Metabolomic Data Analysis

One sample from the pre‐op timepoint was not available for analysis, so that patient's samples were withdrawn. Metabolomic data were loaded into Metaboanalyst 6.0 (www.metaboabalyst.ca) as peak intensities. The data were log‐transformed and mean‐centred scaled before being exported. Principal component analysis (PCA) was performed on metabolomic data, and time‐related patterns were calculated by one‐way repeated measures ANOVA using Tukey's range test to identify interday differences (Aabel 3.0 Gigawiz Ltd).

To compare metabolite expression with physiological or biochemical parameters, the data for each day from all patients were analysed using the biweight‐mid correlation function in the R package (Vienna, Austria) and weighted genome co‐expression network analysis (WGCNA) separately. Correlation coefficients between both modules and individual metabolites with physiological parameters were determined using robust biweight‐mid correlation in the exploratory phase to reduce over‐reliance on outlying values. Correlations of interest were recalculated using Pearson's product moment, as no obvious outliers were identified.

## 3 Results

### 3.1 Patient Demographics

Seventy‐two eligible patients were screened between 19 February 2019 and 28 February 2020; of those, 43 patients were recruited to the study (CONSORT diagram; Figure S1). Thirty‐one patients completed physical assessments up to Day 7 post‐op, and of those, 22 completed follow‐up. Curtailment of the study was caused by the COVID pandemic. Patient demographics, surgical parameters and clinical outcomes are shown in Table S2. Comparing baseline characteristics from the patients who missed follow‐up (*n* = 31) and the group that attended follow‐up (*n* = 22) revealed no differences (data not shown).

### 3.2 Muscle Tissue Outcomes

**FIGURE 1:**
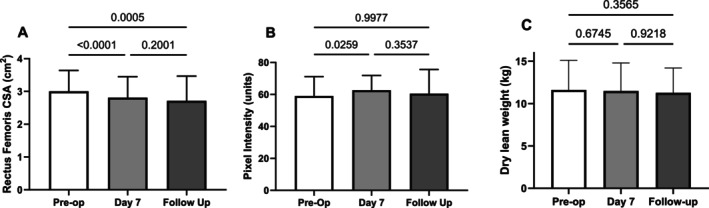
Time course after aortic surgery of indices of muscle bulk and quality. (A) Loss of the rectus femoris cross‐sectional area (RFcsa) by ultrasound from baseline pre‐op (*n* = 31) to Day 7 post‐op (*n* = 31) continued until outpatient follow‐up (*n* = 22: data mean [standard deviation] analysed using Tukey's multiple comparisons test). (B) Muscle damage by ultrasound pixel intensity (units measured by Adobe photoshop) increased post‐operatively at 7 days but recovered to baseline by the time patients returned for follow‐up (data mean [standard deviation] analysed using Tukey's multiple comparisons test). (C) Dry lean weight by bioimpedance showed no change over the period of measurement (data mean [standard deviation] analysed using Tukey's multiple comparisons test).

The patients' RFcsa decreased consistently in the week after surgery as previously, but thereafter the recovery of muscle bulk was variable (Figure 1A). Proportionate mean muscle loss between pre‐op and Day 7 was 6.44% [95% CI 4.21 to 8.68, *n* = 31]; between pre‐op and follow‐up was 9.69% [95% CI 4.92 to 14.96, *n* = 22]; and between Day 7 and follow‐up was 3.60% [95% CI −1.30 to 8.48, *n* = 22]. Separate analysis of the 22 patients completing all timepoints revealed an identical pattern of muscle loss (data not shown). Ultrasound image pixel intensity increased between pre‐op and Day 7, consistent with muscle injury, and returned to baseline at follow‐up (Figure 1B). Dry lean weight, which is heavily influenced by muscle tissue, measured by bioimpedance, tended to decrease progressively throughout the time course but by tiny amounts (Figure 1C). Nutritional intake was not monitored during the study but neither body weight (79.80 [12.49] vs. 78.58 [12.03] kg, mean [SD], *n* = 31 and 22) nor total body water (42.76 [6.22] vs. 42.00 [6.32] kg, mean [SD], *n* = 31 and 22) changed significantly between pre‐operative assessment and follow‐up.

### 3.3 Measures of Strength and Function

**FIGURE 2:**
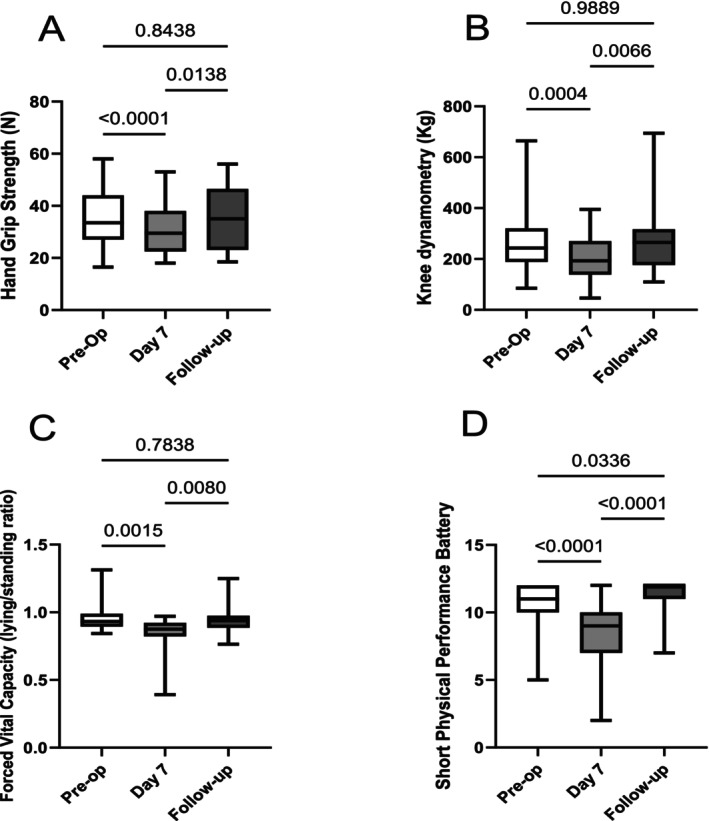
Time course after aortic surgery of measures of muscle strength and function. Handgrip (A); knee extension by dynamometry (B); forced vital capacity ratio lying/standing (C); and Short Physical Performance Battery (SPPB; D) for patients undergoing aortic valve surgery measured pre‐operatively, on Day 7 post‐op and at follow‐up (*n* = 31, 31 and 22). All measures showed transient weakness or loss of function at Day 7, which recovered completely by the time patients returned for follow‐up. Data median (interquartile range) analysed using Tukey's multiple comparisons test.

By contrast to measures of muscle bulk, all the strength and functionality assessments followed the same pattern of a decrease in the first week after surgery (pre‐op to Day 7) followed by a return to baseline (Day 7 to follow‐up; Figure 2A–D). Proportionate strength loss between pre‐op and Day 7 was as follows: for handgrip 8.59% [95% CI 2.86 to 12.88]; for knee extension 17.58% [95% CI 5.90 to 32.56]; for FVC lying/standing 6.73% [95% CI 4.51 to 10.87]; and SPPB 19.08% [95% CI 9.54 to 28.62: all data medians, *n* = 31].

### 3.4 Health‐Related Quality of Life

**FIGURE 3:**
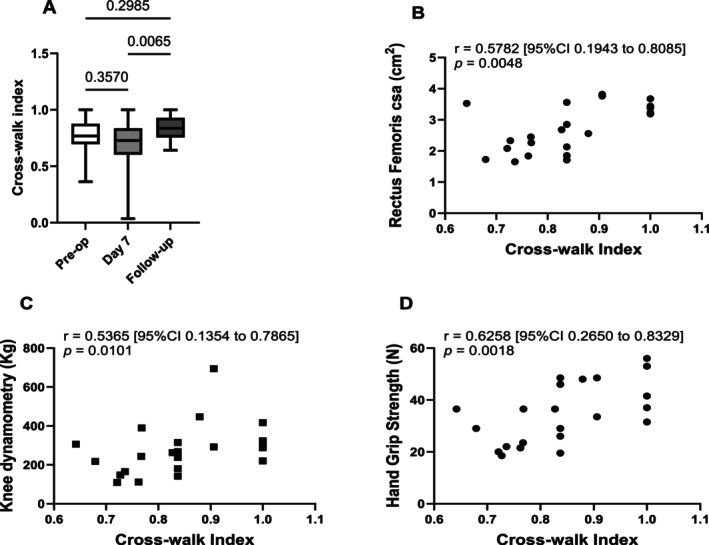
Time course after aortic surgery of measures of health‐related quality of life (cross‐walk index) and correlations with muscle strength and function at follow‐up. (A) Responses to questionnaires answered pre‐operatively, on Day 7 post‐op and at follow‐up (*n* = 31, 31 and 22), showed that the cross‐walk index decreased in the week after surgery and recovered to baseline levels at follow‐up. Data median (interquartile range) analysed using Dunn's multiple comparisons test. (B–D) Cross‐walk index correlated positively with indices of muscle bulk and strength at outpatient follow‐up. Data analysed by Spearman rank correlation: *n* = 19–21.

Unsurprisingly, the cross‐walk index of health‐related quality of life tended to a small decrement in the immediate post‐op period (Day 7; Figure 3A) but recovered to baseline at follow‐up. There was a positive correlation between the cross‐walk index and muscle bulk (RFcsa: *r* = 0.58 [95% CI 0.19 to 0.81] *p* = 0.005; Figure 3B) and strength of knee extension (*r* = 0.54 [95% CI 0.14 to 0.79] *p* = 0.010) and handgrip (*r* = 0.63 [95% CI 0.27 to 0.83] *p* = 0.002; Figure 3C,D).

### 3.5 Mediators of Muscle Homeostasis and Markers of Inflammation

As previously, IGF‐1 plasma concentration decreased immediately after surgery and recovered to baseline by follow‐up (Figure S2A), whereas there was no change in FGF‐21 in this patient cohort across the time course (Figure S2B). By contrast, GDF‐15 was elevated in the week after surgery, returning to baseline at follow‐up (Figure S2C). GDF‐15 has been mooted as a mediator of muscle wasting, and circulating levels were elevated in several chronic conditions. Here, pre‐operative plasma GDF‐15 concentrations correlated negatively with muscle strength, quality and total body function (Table S3), with similar correlations being maintained between plasma concentrations on Day 1 and physical performance at follow‐up (Table S4).

The peri‐operative inflammatory response is illustrated by the profiles of circulating white blood cells and C‐reactive protein (Figure S2D,E). Patients' pre‐op body mass index (BMI) correlated with the subsequent peak (Day 3) circulating CRP (*r* = 0.55, 95% CI 0.23 to 0.76, *p* = 0.001, *n* = 31). Day 3 CRP correlated with patients' pre‐op impedance‐derived fat mass (*r* = 0.39, 95% CI 0.01 to 0.67, *p* = 0.040, *n* = 28) but not the fat‐free mass (*r* = 0.28, 95% CI −0.12 to 0.60, *p* = 0.154, *n* = 28), suggesting that fat tissue may be important in this association. Fat mass correlated with the proportionate loss of muscle mass (RFcsa follow‐up/pre‐op: *r* = −0.55, 95% CI −0.80 to −0.16, *p* = 0.009, *n* = 21) but inversely with the recovery of knee extension strength (*r* = 0.56, 95% CI 0.17 to 0.80, *p* = 0.008, *n* = 21).

### 3.6 Time Course of Circulating Metabolites

PCA showed that the sample timepoints were separable along PC1. Comparing PC1 values, Days 3 and 7 were different from pre‐op and follow‐up and from each other: the latter did not differ from each other (Figure S3). Neither PC1 nor PC2 was different between individuals who wasted and those who did not on any day. The main negative contributors to PC1 were the methylxanthines (metabolites of food and drinks), which reduced with presurgery starvation and returned to normal by follow‐up. Positive contributors to PC1 included nucleotide derivatives, probable markers of tissue turnover. The largest contributor to the latter was 7‐methyl‐guanosine, likely to be derived from the 5′‐methylguanosine caps from mRNA. These metabolites increased after surgery then returned to baseline by follow‐up. Further analysis of a subset of the amino acids and some of their metabolites (isoleucine, leucine, phenylalanine and 3‐hydroxyisovaleric acid) and nucleotide metabolites (N6‐methyl adenosine, N1‐methyl inosine and 7‐methyl‐guanosine) showed that these were all elevated on Day 3 compared with presurgery and returned to baseline by follow‐up consistent with a muscle wasting or tissue injury model, and our previous data where circulating levels of amino acids and nucleotides peaked on Day 3 (Figure S4) [7].

**FIGURE 4:**
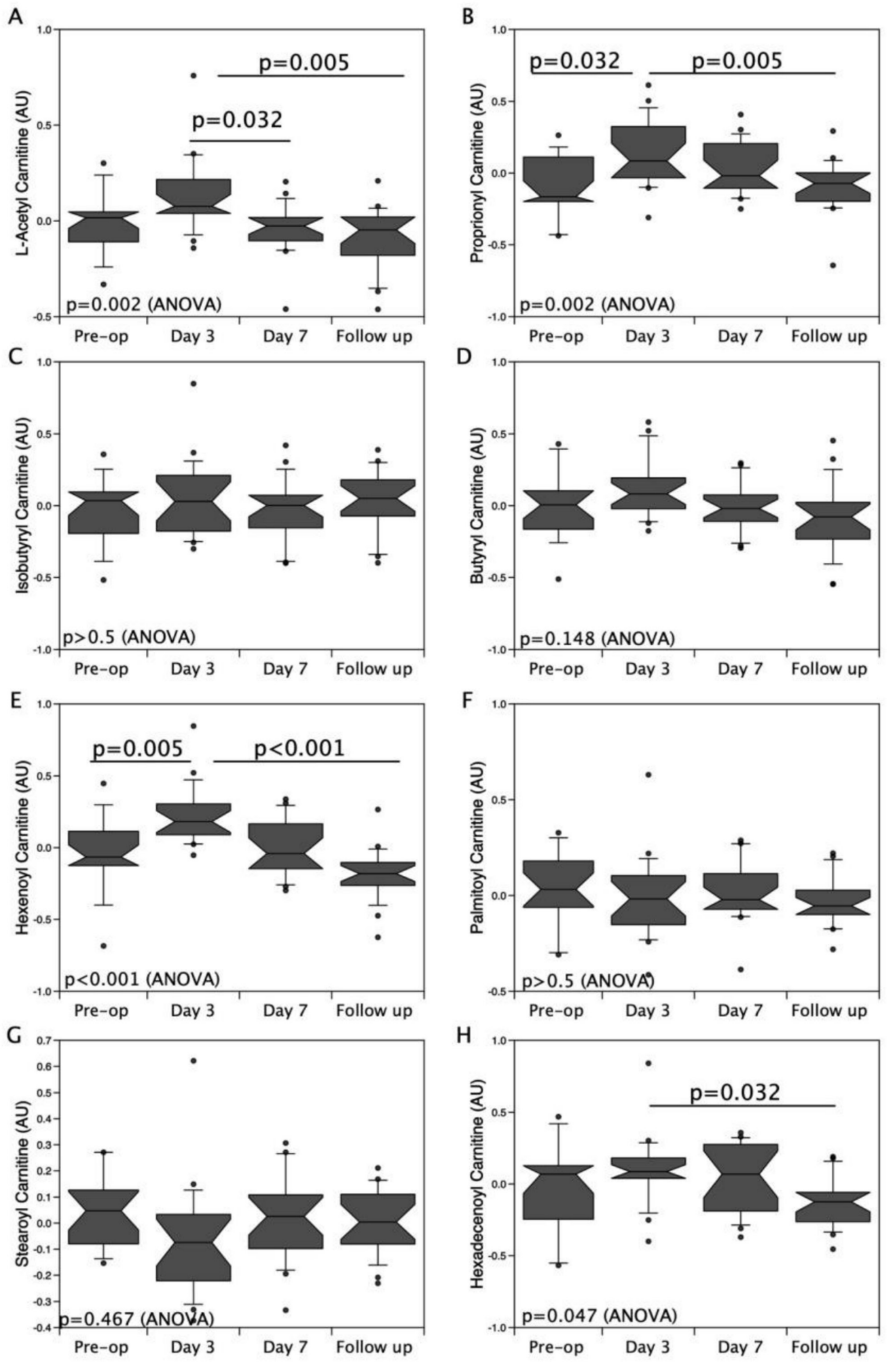
The effect of surgery on short‐ and long‐chain acyl‐carnitines in the circulation of wasting and non‐wasting patients. Short‐chain and dicarboxylate acyl‐carnitines (A–E) measured by reverse phase liquid chromatography mass spectroscopy tended to increase in the days after surgery, whereas long‐chain acyl‐carnitines (F–H) tended to decrease with both returning to baseline values within 7 days (median with interquartile range and outliers at 90% shown as individual points, comparisons by two‐way ANOVA, *n* = 19).

Our previous study showed that short‐chain acyl‐carnitines were increased immediately after surgery before returning to baseline, whereas large acyl‐carnitines were reduced before returning to baseline [7]. Analysis of acyl‐carnitines in this study showed a similar pattern with increased circulating levels of short‐chain acyl‐carnitines 3 days after surgery that returned to baseline by 7 days (Figure 4). Significant reductions in long‐chain acyl‐carnitines were not observed in this study, perhaps because the first timepoint studied was Day 3 rather than Day 1, and because the patients had fewer risk factors, as suggested by a lower pre‐operative EuroScore 2: for this study 1.66% [95% CI 1.23 to 2.21, *n* = 30] compared with 2.21% [95% CI 1.64 to 3.52, *n* = 45: *p* = 0.05] in the previous study [7].

We next investigated correlations of metabolites with proportionate muscle loss (RFcsa at Day 7 and at follow‐up compared with the pre‐operative value) and muscle strength (handgrip and knee extension).

### 3.7 Association of Plasma Metabolites With Rectus Femoris Cross‐Sectional Area Loss

Correlation of the levels of presurgical metabolites with proportionate muscle loss over the 7 days after surgery showed few associations and no obvious pattern other than the inclusion of the two carnitine derivatives, hexaenoyl‐carnitine and hexadecanoyl‐carnitine (Table 1), which were positively associated with the percent of muscle lost. The strongest negative association was with 5‐hydroxyindole sulphate, which decreased after surgery before recovering to baseline and which was lower in those who lost more than 10% RFcsa than in those who lost less than 10% RFcsa, both in this cohort and in our previous study (Figure S5) [7]. Of the presurgical plasma metabolites, octenoyl‐carnitine was strongly associated (*r* = 0.70, *p* < 0.001) with proportionate muscle loss at follow‐up (% RFcsa at follow‐up) suggesting an influence of pre‐operative mitochondrial dysfunction on muscle loss (Table S5) [14].

**TABLE 1:** Top 10 ranked correlation of preoperative plasma metabolites and proportionate muscle loss a week after aortic surgery.

Metabolites (pre‐operative)	Correlation (*r*) with % loss of RFcsa at Day 7	*p* value
5‐hydroxyindole sulphate	−0.625	0.004
2,6‐Dihydroxybenzoic acid	−0.526	0.021
Hexadecenoyl‐carnitine	0.494	0.031
Guanidinobutanoic acid	−0.468	0.043
Hexanoyl‐carnitine	0.459	0.048
Indoxyl sulphate	−0.446	0.055
Azelaic acid	0.439	0.060
Trigonelline	0.428	0.068
Cholic acid, ursocholic acid	−0.426	0.069
Octenoyl‐carnitine	0.406	0.085

*Note:* Correlation coefficients between individual metabolites and rectus femoris cross‐sectional area (RFcsa) change (Day 7/pre‐operative values) were determined using Pearson's product moment after robust biweight‐mid correlation (*n* = 19).

**FIGURE 5:**
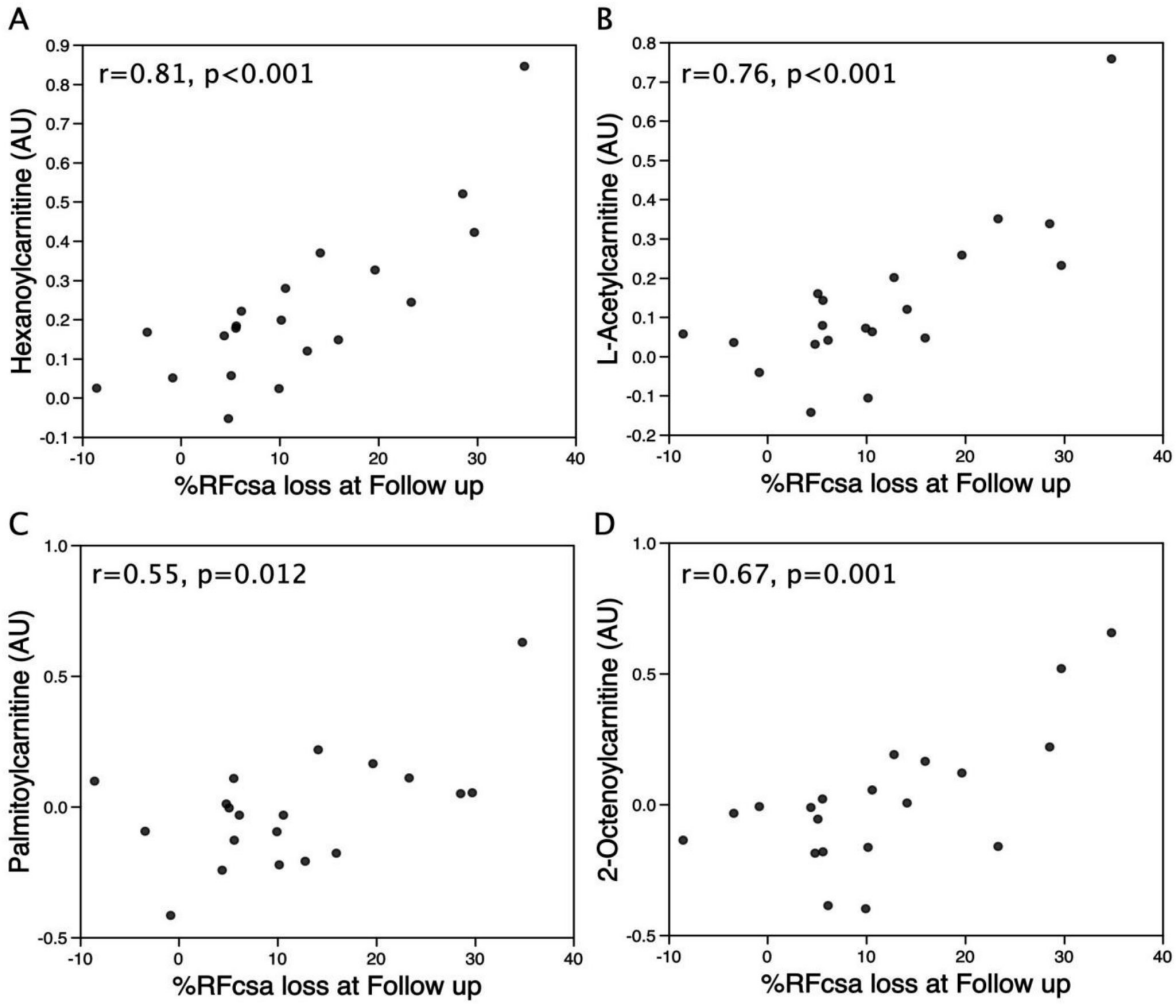
Circulating acyl‐carnitines measured at Day 3 after aortic surgery were associated with proportional loss of muscle mass comparing pre‐operative and follow‐up measurements of rectus femoris cross‐sectional area (RFcsa: cm^2^). Multiple short‐chain acyl‐carnitine markers of mitochondrial dysfunction measured in the plasma at Day 3 post‐operatively predicted proportional muscle loss at follow‐up. Correlations were calculated using Pearson product moment, *n* = 19.

In the Day 3 metabolite data, the tryptophan metabolites 5‐hydroxyindole sulphate and indoxyl sulphate were directly proportional to muscle loss over the first week post‐operatively, suggesting that differences in tryptophan metabolism or nutrition contributed to differences in muscle homeostasis after an acute insult (Table 2). Similarly, comparing the Day 3 metabolites with % RFcsa loss at follow‐up showed significant positive associations of four different acyl‐carnitines, suggesting that peak mitochondrial dysfunction predicted poor muscle recovery and/or sustained muscle wasting (Table 3 and Figures 4 and 5). Similarly, two other tryptophan metabolites, indoxyl sulphate and indoxyl glucuronide, were also among the Day 3 metabolites most closely associated with muscle loss at follow‐up.

**TABLE 2:** Top 10 ranked correlation of Day 3 plasma metabolites and proportionate muscle loss a week after aortic surgery.

Metabolites (Day 3)	Correlation (*r*) with % loss of RFcsa at Day 7	*p* value
5‐hydroxyindole sulphate	−0.666	0.001
Phenol sulphate	−0.612	0.004
Indoxyl sulphate	−0.596	0.006
Malic acid	−0.567	0.009
Glycochenodeoxycholic acid 3‐sulphate	−0.564	0.010
N‐Acetylaspartic acid	−0.517	0.020
Glycocholic acid	−0.504	0.024
N‐acetylglutamic acid	−0.499	0.025
Uric acid	−0.488	0.029
Succinyladenosine	−0.482	0.031

*Note:* Correlation coefficients between individual metabolites and rectus femoris cross‐sectional area (RFcsa) change (Day 7/pre‐operative values) were determined using Pearson's product moment after robust biweight‐mid correlation (*n* = 19).

**TABLE 3:** Top 10 ranked correlation of Day 3 plasma metabolites and proportionate muscle loss at follow‐up after aortic surgery.

Metabolites (Day 3)	Correlation (*r*) with % loss of RFcsa at follow‐up	*p‐*value
Hexanoyl‐carnitine	0.768	< 0.001
L‐Acetyl‐carnitine	0.626	0.003
Sucrose	−0.610	0.004
Sumiki s acid	−0.608	0.004
2‐Octenoyl‐carnitine	0.602	0.005
Indoxyl sulphate	−0.582	0.007
Indoxyl glucuronide	−0.575	0.008
p‐Cresol sulphate	−0.518	0.019
Deoxycholic acid	−0.486	0.030
p‐Hydroxymandelic acid	−0.476	0.034

*Note:* Correlation coefficients between individual metabolites and rectus femoris cross‐sectional area (RFcsa) change (follow‐up/pre‐operative values) were determined using Pearson's product moment after robust biweight‐mid correlation (*n* = 19).

### 3.8 Association of Plasma Metabolites With Measures of Muscle Strength

Relatively few plasma metabolites correlated with proportionate loss of handgrip strength and knee extension over the course of the study (pre‐op/follow‐up), and there was little overlap between correlations with handgrip and knee extension (Tables S6 and S7). The pre‐operative metabolites associating with proportionate loss of handgrip strength up to follow‐up were the tryptophan metabolite, indoxyl glucuronide and the nucleotide breakdown products xanthine and hypoxanthine (Table S6). Days 3 and 7 circulating metabolites associated with proportionate loss of strength included markers of mitochondrial function (citric acid, cis‐aconitic acid and tiglylcarnitine); amino acids and nucleotides and their metabolites (n‐acetylaspartic acid, histidine and succinyladenosine) were associated with proportional loss of handgrip strength (Table S6).

## 4 Discussion

Extending the duration of observation of patients after elective aortic valve surgery with a view to exploring mechanisms of recovery from ICUAW surprisingly showed divergence between muscle bulk and strength at outpatient follow‐up. All measures of strength and functionality (knee extension, handgrip, spirometry and SPPB) had returned to baseline by the time patients returned for follow‐up, but approximately half of the patients had lower values of RFcsa, reflecting quadriceps muscle bulk, compared with Day 7 and baseline, suggesting that the processes causing muscle wasting predominated over that period. Metabolomic analysis of patients' plasma in this study showed similarities to our previous cohort with increased circulating levels of short‐chain acyl‐carnitines 3 days after surgery that returned to baseline by 7 days. Acyl‐carnitines, markers of mitochondrial dysfunction, correlated with adverse muscle outcomes generally, but Day 3 levels in particular correlated strongly with muscle loss (RFcsa) over the period of observation up to outpatient follow‐up.

Both muscle bulk (RFcsa) and strength correlated with health‐related quality of life in the follow‐up period consistent with the expected association between muscle recovery and well‐being. Circulating markers of inflammation (white cell count and CRP) and known modulators of muscle homeostasis (IGF‐1, GDF‐15 and FGF‐21) followed expected peri‐operative profiles, although only plasma GDF‐15 concentration showed promise as a candidate biomarker of acute illness‐related muscle dysfunction. In preliminary observations, there was an association between BMI and fat mass pre‐operatively and subsequent circulating CRP levels. Fat mass correlated positively with loss of muscle bulk (RFcsa) over the course of follow‐up but inversely with loss of strength.

Our findings are significant both for patients undergoing cardiac surgery and as a model of ICUAW. Whilst the long‐term significance of muscle wasting after aortic and cardiac surgery is uncertain, decreased muscle mass immediately before surgery in patients followed up for 8 years after coronary artery bypass surgery correlates with mortality [15]. Hence, targeting muscle health before surgery with prehabilitation and nutrition, or an as yet undiscovered prophylactic drug, or after surgery with similar physical and pharmacological strategies, may improve patient outcomes including long‐term mortality and quality of life.

Critical illness myopathy is a major contributor to post‐ICU syndrome [16]. From a cohort of survivors with ICUAW at ICU discharge, only 38% recovered fully, and 62% had persistent symptoms during a study that followed up patients for up to 10 years [17]. The mechanisms underlying the time course of muscle recovery and the plateau in its function are poorly understood, but mitochondrial content, proteolysis and autophagy had recovered 6 months after ICU admission in one well characterised cohort of survivors [18]. However valuable the latter study is by dint of paired quadriceps muscle biopsies from patients recovering from ICUAW at 7 days and 6 months after ICU discharge, the low patient numbers, heterogeneous population and lack of a premorbid baseline make interpretation difficult and emphasise the potential utility of human models of the sort that we have characterised here. The pathophysiology of longer‐term muscle dysfunction may be determined by satellite cell dysfunction, infiltration by adipocytes or fibroblasts, and endocrine abnormalities (reviewed [19]).

Elevated levels of circulating short‐chain acyl‐carnitines have been associated with sarcopenia of multiple aetiologies [20] and may play a role in muscle wasting by inducing inflammation in muscle cells [21]. Circulating acyl‐carnitine levels reflect cellular accumulation caused by impaired mitochondrial function [14]. Of relevance to the aortic surgery cohort, elevated circulating levels correlated with NT‐pro‐brain natriuretic peptide in heart failure [22], in critical illness with cell‐free plasma mitochondrial DNA [23], and with increased mortality in coronary artery bypass grafting [24]. In the same aortic surgery model, the association of acyl‐carnitines with acute muscle loss suggested a role for mitochondrial dysfunction [7], which we have repeated and extended by demonstrating a strong correlation between Day 3 acyl‐carnitines and muscle wasting that persists after the patient has been discharged from the hospital.

The observation that mitochondrial dysfunction is associated with an increased potential for muscle loss in patients with critical illness is consistent with other studies in animals and humans. In mouse models of sepsis, there is a marked reduction in mitochondrial mass and function as demonstrated by a reduction in metabolic rate [25]. Similarly, survivors of multi‐organ failure had higher levels of PGC1a and better maintenance of complex I activity, indicative of maintained mitochondrial function [26]. In a long‐term rat model of sepsis, reduced complex I activity and ATP depletion were also associated with mortality [27]. Two studies have indicated that mitochondrial dysfunction as a consequence of comorbidities prior to critical illness is associated with muscle loss and weakness in patients. Firstly, in the early phase of critical illness, a greater reduction in muscle ATP levels and loss of mitochondrial function with increased muscle loss occurred in patients with significant comorbidities [28]. Secondly, our study of micro‐(mi)RNAs in aortic surgery patients showed that presurgical miR‐542‐3p was proportional to the amount of muscle lost in patients, and that elevated expression of this miRNA in muscle caused mitochondrial dysfunction by disrupting mitochondrial ribosome formation [9]. That expression of this miRNA was inversely proportional to left ventricular ejection fraction in our aortic surgery patients and to gas transfer in COPD patients, providing a potential mechanism by which the primary disease may promote muscle mitochondrial dysfunction [9, 29].

Circulating concentrations of branched‐chain amino acids, leucine, isoleucine, and the aromatic amino acid tryptophan were reduced in association with sarcopenia in a systematic review of metabolomic studies [30]. In this study, whilst the appearance of the branched‐chain amino acids (leucine and isoleucine) in the circulation on Day 3 after surgery, a stimulus to muscle protein breakdown, was unsurprising, the role of tryptophan metabolites is less easy to explain. However, a metabolite of tryptophan (5‐hydroxyindole sulphate) differentiated patients who lost more than 10% RFcsa (wasters) from non‐wasters, and other metabolites of tryptophan were inversely associated with muscle loss. 5‐hydroxyindole sulphate levels decreased after surgery then returned to baseline during the recovery. The observation that the differences in levels of this metabolite between wasting and non‐wasting patients were re‐established in the recovery period raises the possibility that relevant dietary and genetic factors contribute to the susceptibility of patients to muscle loss following surgery. Although most interest in the sepsis and sarcopenia literature has focused on the kynurenine pathway of tryptophan metabolism, 5‐hydroxyindole is a metabolite of serotonin through the activity of monoamine oxidase and alcohol dehydrogenase [31]. Finally, whilst the association of the serotonin 5‐HT2A receptor with JAK/STAT pathway constituents suggests a potential role in muscle repair [32], our data can only highlight a potential metabolic pathway of interest.

### 4.1 Limitations of the Study

The data presented include a relatively small number of patients from a single centre, all of whom identified as white British. The coincident COVID‐19 pandemic curtailed recruitment, prevented participants from being assessed physically at follow‐up, and delayed biochemical analysis of samples. We have included patients' data for early timepoints who missed attending for follow‐up in person owing to COVID‐19 because the major cause was unbiased (CONSORT diagram; Figure S1). Post hoc power calculations based on the RFcsa data suggest 11 participants per group (22 in total) or 46 participants per group (92 in total) using a paired *t*‐test, significance level (alpha) 0.05 (two‐tailed) and target power 0.8 comparing pre‐operative and follow‐up RFcsa or Day 7 (nadir) and follow‐up RFcsa, respectively.

The cardiac surgery model was first used to investigate muscle dysfunction associated with acute insults and, as such, is a subclinical model of ICUAW. Patients in this study had an average age of 69 years and a significant burden of comorbidities (Table 1) and so, based on pre‐existing mitochondrial dysfunction, would be expected to suffer more wasting and weakness in response to an insult than a previously healthy population [28]. The unexpected continuation of wasting beyond hospital discharge and its association with health‐related quality of life suggests that this phenomenon may be clinically significant, requiring both a longer period of follow‐up and activity monitoring to characterise the population who recover slowly. Finally, the pragmatic study design was associated with lumping into time categories at ‘Follow‐up’. Follow‐up visits occurred at a mean of 73.14 days (standard deviation 21.19, 95% CI 63.74 to 82.53, *n* = 22) with a correlation with proportional loss of RFcsa between pre‐operative and follow‐up of *r* = 0.37, 95% CI −0.071 to 0.69, *p* = 0.087, suggesting that the variation in the interval between hospital discharge and follow‐up cannot account for the variation in long‐term muscle loss.

Trends and associations of the plasma concentrations of IGF‐1 and GDF‐15, and the acyl‐carnitines here are like our previous studies and those of others in similar models. Similarly, the extensive literature supporting the role of GDF‐15 and mitochondrial dysfunction in muscle homeostasis suggests repeatability and biological feasibility [33]. In a pilot study of 142 patients recovering from acute respiratory failure, circulating GDF‐15 levels correlated with poor muscle outcomes [34]. In the same human cardiac surgery model used here, GDF‐15 remained elevated for longer in patients who lost more than 10% of their quadriceps muscle bulk [35], caused wasting when over‐expressed in mouse tibialis anterior muscles [36] and suppressed the expression of micro‐RNAs to increase myoblast sensitivity to transforming growth factor beta (TGFβ)‐1 [35]. GDF‐15 inhibits appetite by activating the GFRAL receptor in the hippocampus [37] also binding and signalling through CD48 in regulatory T cells [38]. We have shown that GDF‐15 increased TGF‐β‐activated kinase 1 (TAK1) activity in cultured myoblasts independent of a SMAD response [39], suggesting that GDF‐15 can signal directly in the muscle. Independence from the anorexigenic effects of GDF‐15 are supported by its association with muscle size and function, but not BMI in COPD and pulmonary arterial hypertension. However, the signalling mechanism in muscle remains to be fully established. Finally, circulating GDF‐15 was elevated in patients and preclinical models of primary mitochondrial myopathy, and administration of a blocking antibody was associated with an improved muscle phenotype in mice expressing a proofreading‐deficient version of the mitochondrial DNA polymerase gamma, leading to an increased rate of mutations in mitochondrial DNA [40].

By contrast, the relationship between pre‐operative BMI/fat mass and peak CRP in this context is more tenuous and would need to be confirmed in a larger population, ideally with clinical outcomes. However, it is well established that obesity is associated with an elevated CRP; for example, in a large cohort of smokers, the influence of obesity over CRP predominated [41]. Also, adipose tissue is a source of pro‐inflammatory cytokines, CRP, adipokines and exerts a strong influence over insulin secretion and metabolism, so that it is equipped to influence systemic inflammation and muscle homeostasis (reviewed [42]).

In conclusion, our data indicate that in response to uncomplicated aortic surgery, there is a short‐term loss of muscle strength but a longer‐term tendency for muscle wasting, which is associated with poorer quality of life after hospital discharge. The extent of loss of muscle is associated with markers of mitochondrial dysfunction.

## Ethics Statement

All participants gave their informed consent prior to their inclusion in the study. Ethical approval was secured from the National Research Ethics Committee (18/LO/1618): The study was registered with clinicaltrials.gov (NCT03714399). The study was carried out in accordance with the ethical standards laid down in the 1964 Declaration of Helsinki and its later amendments.

## Conflicts of Interest

The authors declare no conflicts of interest.

## Supporting information


**Table S1:** Plasma metabolites detected by liquid chromatography mass spectroscopy.
**Table S2:** Baseline characteristics and clinical outcomes. Data presented as either number (n), frequency (%), mean [SD], or median [IQR] and range. Key: M, male; cm, centimetres; kg, kilograms, μmol/l, micromole per litre; m, metres; ml, millilitres; min, minute; IQR, interquartile range; WHO, World Health Organisation; LVEF, left ventricular ejection fraction; AVR, aortic valve replacement; CABG, coronary artery bypass graft; ICU, intensive care unit.
**Table S3:** Correlation (Spearman analysis) between pre‐operative circulating GDF‐15 concentration and patient age, pre‐operative handgrip (HG), knee extension (KE), muscle quality (rectus femoris pixel intensity: PI) and short physical performance battery (SPPB).
**Table S4:** Correlation (Spearman analysis) between Day 1 circulating GDF‐15 concentration and handgrip (HG), knee extension (KE), muscle quality (rectus femoris pixel intensity: PI) and short physical performance battery (SPPB) at follow‐up (FU).
**Table S5:** Top 10 ranked correlation of pre‐operative plasma metabolites and proportionate muscle loss at follow‐up after aortic surgery. Correlation coefficients between individual metabolites and rectus femoris cross‐sectional area (RFcsa) change (follow‐up/pre‐operative values) were determined using Pearson's product moment after robust biweight‐mid correlation (*n* = 19).
**Table S6:** Top 10 ranked correlation of pre‐operative plasma metabolites and proportionate handgrip strength at follow‐up after aortic surgery. Correlation coefficients between individual metabolites and handgrip strength change (follow‐up/pre‐operative values) were determined using Pearson's product moment after robust biweight‐mid correlation (*n* = 19).
**Table S7:** Top 10 ranked correlation of Day 3 plasma metabolites and proportionate handgrip strength at follow‐up after aortic surgery. Correlation coefficients between individual metabolites and handgrip strength change (follow‐up/pre‐operative values) were determined using Pearson's product moment after robust biweight‐mid correlation (*n* = 19).
**Table S8:** Top 10 ranked correlation of pre‐operative plasma metabolites and knee extension measured pre‐operatively, at Day 7 and at follow‐up. Correlation coefficients between individual metabolites and knee extension strength were determined using Pearson's product moment after robust biweight‐mid correlation (*n* = 19).

**Data S1:** Supplementary information.

**Figure S1:** Modified CONSORT diagram. CVA, cerebral vascular accident; DNA, did not attend.
**Figure S2:** Time course after aortic surgery of circulating mediators of muscle homeostasis and markers of inflammation. A, B, C: Plasma levels of insulin‐like growth factor (IGF)‐1, fibroblast growth factor (FGF)‐21 and growth and differentiation factor (GDF)‐15 were quantified by ELISA. D: The white cell count was derived from Routine full blood count Coulter counter data. E: C‐reactive protein measured in the clinical laboratory. Data analysed using Dunn's multiple comparisons against pre‐op baseline (Day 0: median [IQR, 95% CI], *n* = 22–30).
**Figure S3:** The effect of aortic surgery on patients' plasma metabolites. A: Principal component analysis was carried out using all available metabolite data following normalisation. Scatter plot of PC1 and PC2 for all samples shows marked change along PC1 from Days 1 to 3 with a small return towards pre‐op values on Day 7. Follow‐up is indistinguishable from Day 0. B: Median PC1 values for each day were plotted with interquartile range and outliers at 90% shown as individual points. There was a marked increase in PC1 between Days 0 and 3 that returned to baseline at follow‐up. C: Median PC2 values for each day are plotted with interquartile range and outliers at 90% shown as individual points. PC2 did not vary significantly over the time course, comparisons by two‐way ANOVA comparing the effect of time on plasma metabolites throughout, *n* = 19.
**Figure S4:** Time course after aortic surgery of circulating amino acids (A to D) and nucleotide metabolites (E to H) from patients' plasma. Plasma amino acids and some of their metabolites (A–D: isoleucine, leucine, phenylalanine and 3‐hydroxyisovaleric acid) and nucleotide metabolites (E–H: N6‐methyl adenosine, N1‐methyl inosine and 7‐methyl‐guanosine) showed that these were all elevated on Day 3 compared with presurgery and returned to baseline by follow‐up (median with interquartile range and outliers at 90% shown as individual points, comparisons by two‐way ANOVA, *n* = 19).
**Figure S5:** Time course after aortic surgery of the tryptophan metabolite 5‐hydroxyindole sulphate (arbitrary units: B and D) and the correlation of pre‐operative levels with proportionate muscle loss at Day 7 post‐operatively (rectus femoris cross‐sectional area RFcsa: A and C) from two cohorts of patients' plasma. Identical analyses were performed in similar patient cohorts: the current (A, B; *n* = 19) and previously published data (C, D; *n* = 20). Correlations were calculated using Pearson product moment. 5‐hydroxyindole sulphate was measured by reverse phase liquid chromatography mass spectroscopy and expressed as arbitrary units (median with interquartile range and outliers at 90% shown as individual points, comparisons by two‐way ANOVA). B and D: 5‐hydroxyindoyle sulphate decreased after surgery before recovering to baseline. Lower concentrations were measured in those who lost more than 10% RFcsa (solid symbols) than those that lost less than 10% RFcsa (open symbols).

## References

[1] Z. A. Puthucheary , J. Rawal , M. McPhail , et al., “Acute Skeletal Muscle Wasting in Critical Illness,” Journal of the American Medical Association 310 (2013): 1591–1600. 24108501 10.1001/jama.2013.278481

[2] M. S. Herridge , C. M. Tansey , A. Matte , et al., “Functional Disability 5 Years After Acute Respiratory Distress Syndrome,” New England Journal of Medicine 364 (2011): 1293–1304. 21470008 10.1056/NEJMoa1011802

[3] S. A. Bloch , A. V. Donaldson , A. Lewis , et al., “MiR‐181a: A Potential Biomarker of Acute Muscle Wasting Following Elective High‐Risk Cardiothoracic Surgery,” Critical Care 19 (2015): 147. 25888214 10.1186/s13054-015-0853-5 PMC4403779

[4] R. Paul , J. Lee , A. V. Donaldson , et al., “miR‐422a Suppresses SMAD4 Protein Expression and Promotes Resistance to Muscle Loss,” Journal of Cachexia, Sarcopenia and Muscle 9 (2018): 119–128. 28984049 10.1002/jcsm.12236 PMC5803610

[5] A. Lewis , J. Y. Lee , A. V. Donaldson , et al., “Increased Expression of H19/miR‐675 Is Associated With a Low Fat‐Free Mass Index in Patients With COPD,” Journal of Cachexia, Sarcopenia and Muscle 7 (2016): 330–344. 27239417 10.1002/jcsm.12078 PMC4863928

[6] P. R. Kemp , M. Griffiths , and M. I. Polkey , “Muscle Wasting in the Presence of Disease, Why Is It So Variable?,” Biological Reviews of the Cambridge Philosophical Society 94 (2019): 1038–1055. 30588725 10.1111/brv.12489

[7] P. R. Kemp , R. Paul , A. C. Hinken , D. Neil , A. Russell , and M. J. Griffiths , “Metabolic Profiling Shows Pre‐Existing Mitochondrial Dysfunction Contributes to Muscle Loss in a Model of ICU‐Acquired Weakness,” Journal of Cachexia, Sarcopenia and Muscle 11 (2020): 1321–1335. 32677363 10.1002/jcsm.12597 PMC7567140

[8] X. Chen , Y. Ji , R. Liu , et al., “Mitochondrial Dysfunction: Roles in Skeletal Muscle Atrophy,” Journal of Translational Medicine 21 (2023): 503. 37495991 10.1186/s12967-023-04369-z PMC10373380

[9] R. F. Garros , R. Paul , M. Connolly , et al., “MicroRNA‐542 Promotes Mitochondrial Dysfunction and SMAD Activity and Is Elevated in Intensive Care Unit‐Acquired Weakness,” American Journal of Respiratory and Critical Care Medicine 196 (2017): 1422–1433. 28809518 10.1164/rccm.201701-0101OC PMC5736972

[10] J. M. Seymour , K. Ward , P. S. Sidhu , et al., “Ultrasound Measurement of Rectus Femoris Cross‐Sectional Area and the Relationship With Quadriceps Strength in COPD,” Thorax 64 (2009): 418–423. 19158125 10.1136/thx.2008.103986

[11] S. A. Bloch , J. Y. Lee , S. J. Wort , M. I. Polkey , P. R. Kemp , and M. J. Griffiths , “Sustained Elevation of Circulating Growth and Differentiation Factor‐15 and a Dynamic Imbalance in Mediators of Muscle Homeostasis Are Associated With the Development of Acute Muscle Wasting Following Cardiac Surgery,” Critical Care Medicine 41 (2013): 982–989. 23328263 10.1097/CCM.0b013e318274671b

[12] M. S. Stock and B. J. Thompson , “Echo Intensity as an Indicator of Skeletal Muscle Quality: Applications, Methodology, and Future Directions,” European Journal of Applied Physiology 121 (2021): 369–380. 33221942 10.1007/s00421-020-04556-6

[13] J. M. Guralnik , E. M. Simonsick , L. Ferrucci , et al., “A Short Physical Performance Battery Assessing Lower Extremity Function: Association With Self‐Reported Disability and Prediction of Mortality and Nursing Home Admission,” Journal of Gerontology 49 (1994): M85–M94. 8126356 10.1093/geronj/49.2.m85

[14] M. R. McGill , F. Li , M. R. Sharpe , et al., “Circulating Acylcarnitines as Biomarkers of Mitochondrial Dysfunction After Acetaminophen Overdose in Mice and Humans,” Archives of Toxicology 88 (2014): 391–401. 23979652 10.1007/s00204-013-1118-1 PMC3946727

[15] S. H. Lee , J. Jo , J. H. Yang , et al., “Clinical Impact of Sarcopenia Screening on Long‐Term Mortality in Patients Undergoing Coronary Bypass Grafting,” Journal of Cachexia, Sarcopenia and Muscle 15, no. 6 (2024): 2842–2851. 39513369 10.1002/jcsm.13645 PMC11634471

[16] D. Intiso , A. M. Centra , M. Bartolo , M. T. Gatta , M. Gravina , and F. Di Rienzo , “Recovery and Long Term Functional Outcome in People With Critical Illness Polyneuropathy and Myopathy: A Scoping Review,” BMC Neurology 22 (2022): 50. 35148710 10.1186/s12883-022-02570-z PMC8831873

[17] C. H. Meyer‐Friessem , N. M. Malewicz , S. Rath , et al., “Incidence, Time Course and Influence on Quality of Life of Intensive Care Unit‐Acquired Weakness Symptoms in Long‐Term Intensive Care Survivors,” Journal of Intensive Care Medicine 36 (2021): 1313–1322. 32799703 10.1177/0885066620949178

[18] C. Dos Santos , S. N. Hussain , S. Mathur , et al., “Mechanisms of Chronic Muscle Wasting and Dysfunction After an Intensive Care Unit Stay. A Pilot Study,” American Journal of Respiratory and Critical Care Medicine 194 (2016): 821–830. 27058306 10.1164/rccm.201512-2344OC

[19] Y. F. N. Boelens , M. Melchers , and A. R. H. van Zanten , “Poor Physical Recovery After Critical Illness: Incidence, Features, Risk Factors, Pathophysiology, and Evidence‐Based Therapies,” Current Opinion in Critical Care 28 (2022): 409–416. 35796071 10.1097/MCC.0000000000000955 PMC9594146

[20] J. Luo , J. Li , W. Wang , R. Zhang , and D. Zhang , “Identifying the Shared Metabolite Biomarkers and Potential Intervention Targets for Multiple Sarcopenia‐Related Phenotypes,” International Journal of Molecular Sciences 25 (2024): 12310. 39596375 10.3390/ijms252212310 PMC11594328

[21] J. M. Rutkowsky , T. A. Knotts , K. D. Ono‐Moore , et al., “Acylcarnitines Activate Proinflammatory Signaling Pathways,” American Journal of Physiology. Endocrinology and Metabolism 306 (2014): E1378–E1387. 24760988 10.1152/ajpendo.00656.2013 PMC4059985

[22] R. M. Gueant Rodriguez , R. Spada , S. Pooya , et al., “Homocysteine Predicts Increased NT‐Pro‐BNP Through Impaired Fatty Acid Oxidation,” International Journal of Cardiology 167 (2013): 768–775. 22459404 10.1016/j.ijcard.2012.03.047

[23] P. I. Johansson , K. Nakahira , A. J. Rogers , et al., “Plasma Mitochondrial DNA and Metabolomic Alterations in Severe Critical Illness,” Critical Care 22 (2018): 360. 30594224 10.1186/s13054-018-2275-7 PMC6310975

[24] A. A. Shah , D. M. Craig , J. K. Sebek , et al., “Metabolic Profiles Predict Adverse Events After Coronary Artery Bypass Grafting,” Journal of Thoracic and Cardiovascular Surgery 143 (2012): 873–878. 22306227 10.1016/j.jtcvs.2011.09.070 PMC3324120

[25] M. Singer , “The Role of Mitochondrial Dysfunction in Sepsis‐Induced Multi‐Organ Failure,” Virulence 5 (2014): 66–72. 24185508 10.4161/viru.26907 PMC3916385

[26] J. E. Carre , J. C. Orban , L. Re , et al., “Survival in Critical Illness Is Associated With Early Activation of Mitochondrial Biogenesis,” American Journal of Respiratory and Critical Care Medicine 182 (2010): 745–751. 20538956 10.1164/rccm.201003-0326OC PMC2949402

[27] D. Brealey , S. Karyampudi , T. S. Jacques , et al., “Mitochondrial Dysfunction in a Long‐Term Rodent Model of Sepsis and Organ Failure,” American Journal of Physiology. Regulatory, Integrative and Comparative Physiology 286 (2004): R491–R497. 14604843 10.1152/ajpregu.00432.2003

[28] Z. A. Puthucheary , R. Astin , M. J. W. McPhail , et al., “Metabolic Phenotype of Skeletal Muscle in Early Critical Illness,” Thorax 73 (2018): 926–935. 29980655 10.1136/thoraxjnl-2017-211073

[29] R. Farre‐Garros , J. Y. Lee , S. A. Natanek , et al., “Quadriceps miR‐542‐3p and ‐5p Are Elevated in COPD and Reduce Function by Inhibiting Ribosomal and Protein Synthesis,” Journal of Applied Physiology 126 (2019): 1514–1524. 30676868 10.1152/japplphysiol.00882.2018 PMC6551227

[30] M. Dai , T. Lin , J. Yue , and L. Dai , “Signatures and Clinical Significance of Amino Acid Flux in Sarcopenia: A Systematic Review and Meta‐Analysis,” Frontiers in Endocrinology 12 (2021): 725518. 34589057 10.3389/fendo.2021.725518 PMC8473793

[31] M. Kanova and P. Kohout , “Tryptophan: A Unique Role in the Critically Ill,” International Journal of Molecular Sciences 22 (2021): 11714. 34769144 10.3390/ijms222111714 PMC8583765

[32] I. Guillet‐Deniau , A. F. Burnol , and J. Girard , “Identification and Localization of a Skeletal Muscle Secrotonin 5‐HT2A Receptor Coupled to the Jak/STAT Pathway,” Journal of Biological Chemistry 272 (1997): 14825–14829. 9169451 10.1074/jbc.272.23.14825

[33] B. H. Wang , M. Y. Qi , Z. Yang , G. L. He , and S. Y. Meng , “Growth Differentiation Factor‐15 as a Biomarker for Intensive Care Unit‐Acquired Weakness: A Meta‐Analysis,” Frontiers in Medicine 12 (2025): 1486361. 39950128 10.3389/fmed.2025.1486361 PMC11821601

[34] B. J. Rosenberg , M. Hirano , C. M. Quinzii , et al., “Growth Differentiation Factor‐15 as a Biomarker of Strength and Recovery in Survivors of Acute Respiratory Failure,” Thorax 74 (2019): 1099–1101. 31534031 10.1136/thoraxjnl-2019-213621 PMC7043788

[35] S. A. Bloch , J. Y. Lee , T. Syburra , et al., “Increased Expression of GDF‐15 May Mediate ICU‐Acquired Weakness by Down‐Regulating Muscle MicroRNAs,” Thorax 70 (2015): 219–228. 25516419 10.1136/thoraxjnl-2014-206225 PMC4345798

[36] M. S. Patel , J. Lee , M. Baz , et al., “Growth Differentiation Factor‐15 Is Associated With Muscle Mass in Chronic Obstructive Pulmonary Disease and Promotes Muscle Wasting In Vivo,” Journal of Cachexia, Sarcopenia and Muscle 7 (2016): 436–448. 27239406 10.1002/jcsm.12096 PMC4864181

[37] P. J. Emmerson , F. Wang , Y. Du , et al., “The Metabolic Effects of GDF15 Are Mediated by the Orphan Receptor GFRAL,” Nature Medicine 23 (2017): 1215–1219. 10.1038/nm.4393 28846098

[38] Z. Wang , L. He , W. Li , et al., “GDF15 Induces Immunosuppression via CD48 on Regulatory T Cells in Hepatocellular Carcinoma,” Journal for Immunotherapy of Cancer 9 (2021): e002787. 34489334 10.1136/jitc-2021-002787 PMC8422483

[39] B. E. Garfield , A. Crosby , D. Shao , et al., “Growth/Differentiation Factor 15 Causes TGFβ‐Activated Kinase 1‐Dependent Muscle Atrophy in Pulmonary Arterial Hypertension,” Thorax 74 (2019): 164–176. 30554141 10.1136/thoraxjnl-2017-211440 PMC6467240

[40] S. E. Flaherty, 3rd , L. Song , B. Albuquerque , et al., “GDF15 Neutralization Ameliorates Muscle Atrophy and Exercise Intolerance in a Mouse Model of Mitochondrial Myopathy,” Journal of Cachexia, Sarcopenia and Muscle 16 (2025): e13715. 39976232 10.1002/jcsm.13715 PMC11840706

[41] S. Gallus , A. Lugo , P. Suatoni , et al., “Effect of Tobacco Smoking Cessation on C‐Reactive Protein Levels in A Cohort of Low‐Dose Computed Tomography Screening Participants,” Scientific Reports 8 (2018): 12908. 30150729 10.1038/s41598-018-29867-9 PMC6110802

[42] C. J. Smith , T. A. Perfetti , A. W. Hayes , and S. C. Berry , “Obesity as a Source of Endogenous Compounds Associated With Chronic Disease: A Review,” Toxicological Sciences 175 (2020): 149–155. 32207534 10.1093/toxsci/kfaa042

