# Supplementary material for: Mitochondrial Dysfunction Contributes to Sustained Muscle Loss After Cardiac Surgery: A Prospective Observational Study
Source: J Cachexia Sarcopenia Muscle. 2025 Aug 21;16(4):e70051. doi: 10.1002/jcsm.70051 (PMC12370447; doi:10.1002/jcsm.70051)
Supplement: Supplementary file 1 — Table S1: Plasma metabolites detected by liquid chromatography mass spectroscopy. Table S2: Baseline characteristics and clinical outcomes. Data presented as either number (n), frequency (%), mean [SD], or median [IQR] and range. Key: M, male; cm, centimetres; kg, kilograms, μmol/l, micromole per litre; m, metres; ml, millilitres; min, minute; IQR, interquartile range; WHO, World Health Organisation; LVEF, left ventricular ejection fraction; AVR, aortic valve replacement; CABG, coronary artery bypass graft; ICU, intensive care unit. Table S3: Correlation (Spearman analysis) between pre‐operative circulating GDF‐15 concentration and patient age, pre‐operative handgrip (HG), knee extension (KE), muscle quality (rectus femoris pixel intensity: PI) and short physical performance battery (SPPB). Table S4: Correlation (Spearman analysis) between Day 1 circulating GDF‐15 concentration and handgrip (HG), knee extension (KE), muscle quality (rectus femoris pixel intensity: PI) and short physical performance battery (SPPB) at follow‐up (FU). Table S5: Top 10 ranked correlation of pre‐operative plasma metabolites and proportionate muscle loss at follow‐up after aortic surgery. Correlation coefficients between individual metabolites and rectus femoris cross‐sectional area (RFcsa) change (follow‐up/pre‐operative values) were determined using Pearson's product moment after robust biweight‐mid correlation (n = 19). Table S6: Top 10 ranked correlation of pre‐operative plasma metabolites and proportionate handgrip strength at follow‐up after aortic surgery. Correlation coefficients between individual metabolites and handgrip strength change (follow‐up/pre‐operative values) were determined using Pearson's product moment after robust biweight‐mid correlation (n = 19). Table S7: Top 10 ranked correlation of Day 3 plasma metabolites and proportionate handgrip strength at follow‐up after aortic surgery. Correlation coefficients between individual metabolites and handgrip strength change [file JCSM-16-e70051-s001.docx]

**SUPPLEMENTARY INFORMATION**

**Mitochondrial dysfunction contributes to sustained muscle loss after cardiac surgery a prospective observational study**

*Ashley N. Thomas^1^, Antonis Kalakoutas^4^, Martin Yates^1^, John Yap^1^, *Julie Sanders^1,2^, *Paul Kemp ^3^, and *Mark J.D. Griffiths^1, 3^*

1. Barts Heart Centre, St Bartholomew's Hospital, Barts NHS Trust, London, UK
2. Florence Nightingale Faculty of Nursing, Midwifery and Palliative Care, Kings College, London, UK
3. National Heart and Lung Institute, Imperial College, London, UK
4. Guy’s and St Thomas’ NHS Trust, London, UK

* these authors made equal contributions

**Short Title:** Mitochondrial dysfunction increases muscle atrophy

**Key Words:** muscle wasting; muscle recovery; aortic surgery; mitochondrial dysfunction; metabolomics; ICU acquired weakness

**Corresponding author:** Mark. J. Griffiths

St Bartholomew's Hospital

Critical Care Unit (6A)

Barts NHS Trust

EC1A 7BE

m.griffiths@ic.ac.uk

**Figure legends (Supplementary)**

**Figure S1: Modified CONSORT diagram**

 CVA, cerebral vascular accident; DNA, did not attend.

**Figure S2: Time course after aortic surgery of circulating mediators of muscle homeostasis and markers of inflammation**

A, B, C: Plasma levels of insulin-like growth factor (IGF)-1, fibroblast growth factor (FGF)-21 and growth and differentiation factor (GDF)-15 were quantified by ELISA. D: the white cell count was derived from Routine full blood count Coulter counter data. E: C-reactive protein measured in the clinical laboratory. Data analysed using Dunn’s multiple comparisons against pre-op baseline (day 0: median [IQR, 95%CI], n=22-30).

**Figure S3: The effect of aortic surgery on patients’ plasma metabolites**

A: Principal component analysis was carried out using all available metabolite data following normalisation. Scatter plot of PC1 and PC2 for all samples shows marked change along PC1 from day 1 to day 3 with a small return towards pre-op values on day 7. Follow-up is indistinguishable from day 0. B: median PC1 values for each day were plotted with interquartile range and outliers at 90% shown as individual points. There was a marked increase in PC1 between day 0 and 3 that returned to baseline at follow-up. C: median PC2 values for each day are plotted with interquartile range and outliers at 90% shown as individual points. PC2 did not vary significantly over the time course, comparisons by 2-way ANOVA comparing the effect of time on plasma metabolites throughout, n=19.

**Figure S4: Time course after aortic surgery of circulating amino acids (A to D) and nucleotide metabolites (E to H) from patients’ plasma**

*Plasma* amino acids and some of their metabolites (A-D: isoleucine, leucine, phenylalanine, *3-hydroxyisovaleric acid*) and nucleotide metabolites (E-H: N6-methyl adenosine, N1-methyl inosine and 7-methyl-guanosine) showed that these were all elevated on day 3 compared with pre-surgery and returned to baseline by follow-up (median with interquartile range and outliers at 90% shown as individual points, comparisons by 2-way ANOVA, n=19).

**Figure S5: Time course after aortic surgery of the tryptophan metabolite 5-hydroxyindole sulphate (arbitrary units: B and D) and the correlation of pre-operative levels with proportionate muscle loss at day 7 post-operatively (rectus femoris cross-sectional area RFcsa: A and C) from two cohorts of patients’ plasma**

Identical analyses were performed in similar patient cohorts: the current (A, B; n=19) and previously published data (C, D; n=20). Correlations were calculated using Pearson product moment. 5-hydroxyindole sulphate was measured by reverse phase liquid chromatography mass spectroscopy and expressed as arbitrary units (median with interquartile range and outliers at 90% shown as individual points, comparisons by 2-way ANOVA). B and D: 5-hydroxyindoyle sulphate decreased after surgery before recovering to baseline. Lower concentrations were measured in those who lost more than 10% RFcsa (solid symbols) than those that lost less than 10% RFcsa (open symbols).

| 1-Methyladenosine |
| --- |
| 1-Methylpiperidine-2-carboxylic acid (N-methyl pipecolic acid) |
| 1-Methyluric acid |
| 1,1-Dimethylbiguanide (Metformin) |
| 1,3,7-Trimethyluric acid |
| 1,7-Dimethyluric acid |
| 2-Furoylglycine |
| 2-hydroxy-3-methylbutyric acid |
| 2-Octenoylcarnitine CAR(8:1) |
| 2,6-Dihydroxybenzoic acid |
| 3-(3-hydroxyphenyl)-3-hydroxypropionic acid (HPHPA) |
| 3-Carboxy-4-methyl-5-propyl-2-furanpropionic acid (CMPF) |
| 3-Hydroxy-3-methylglutaric acid |
| 3-Hydroxyhippuric acid |
| 3-hydroxyisovaleric acid |
| 3-Methoxytyrosine |
| 3-Methyl-2-oxovaleric acid |
| 3-Methylxanthine |
| 3,7-Dimethyluric acid |
| 4-Ethylphenylsulfate |
| 4-Guanidinobutanoic acid |
| 4-Hydroxyhippuric acid |
| 4-Hydroxyphenyllactic acid |
| 5-hydroxyindole sulfate |
| 5'-Methylthioadenosine |
| 7-Methylguanine |
| 7-Methylguanosine |
| 7-Methylxanthine |
| Acetaminophen |
| Acetaminophen Glucuronide |
| Acetaminophen Sulfate |
| Adipic acid |
| Azelaic Acid |
| Betaine |
| Butyrylcarnitine CAR(4:0) |
| Caffeine |
| Cholic acid \| Ursocholic acid |
| Cis-aconitic acid |
| Citraconic acid |
| Citric acid |
| Cortisone |
| Cotinine |
| Dehydroepiandrosterone Sulfate |
| Deoxycholic acid |
| Dopamine 3-O-sulfate |
| Glycochenodeoxycholic acid-3-Sulfate |
| Glycocholic acid |
| Glycodeoxycholic acid |
| Glycodeoxycholic acid-3-Sulfate |
| Glycolithocholic acid-3-Sulfate |
| Glycoursodeoxycholic acid |
| Glycoursodeoxycholic acid-3-Sulfate |
| Hexadecenoylcarnitine CAR (16:1) |
| Hexanoylcarnitine CAR(6:0) |
| Hippuric acid |
| Histidine |
| Homocitrulline |
| Hypoxanthine |
| Imidazolepropionic acid |
| Indole-3-acetic acid |
| Indolelactic acid |
| Indoxyl glucuronide |
| Indoxyl sulfate |
| Isobutyrylcarnitine CAR(4:0) |
| Isocitric acid |
| Isoleucine |
| Ketoleucine |
| Kynurenic acid |
| Kynurenine |
| L-Acetylcarnitine CAR(2:0) |
| L-Ascorbic acid 2-sulfate |
| Leucine |
| Leucylleucine |
| Malic acid |
| N-a-Acetyl-L-arginine |
| N-acetyl-L-carnosine |
| N-Acetylaspartic acid |
| N-acetylglutamic acid |
| N-Acetylisoputreanine |
| N-Acetylisoputreanine-gamma-lactam |
| N-Acetylneuraminic acid |
| N-methyl-L-proline |
| N1-methylinosine |
| N2,N2-Dimethylguanosine |
| N6-Acetyl-L-lysine |
| N6-Methyladenosine |
| Niacinamide |
| O-Sulfotyrosine |
| p-Cresol glucuronide |
| p-Cresol sulfate |
| p-Hydroxymandelic acid |
| Palmitoylcarnitine CAR(16:0) |
| Pantothenic acid |
| Paraxanthine |
| Phenol sulfate |
| Phenylacetylglutamine |
| Phenylalanine |
| Phenylalanyltryptophan |
| Phenyllactic acid |
| Pipecolic acid |
| Pregnanediol-3-glucuronide |
| Pregnenolone sulfate |
| Proline betaine |
| Prolylhydroxyproline |
| Propionylcarnitine CAR(3:0) |
| Pseudouridine |
| Pyralline |
| Pyroglutamic acid |
| Quinic acid |
| Riboflavin (vit B2) |
| Saccharin |
| Salicylic acid |
| Salicyluric acid |
| Serotonin |
| Stearoylcarnitine CAR(18:0) |
| Succinic acid |
| Succinyladenosine |
| Sucrose |
| Sumiki's acid |
| Symmetric \| Asymmetric Dimethylarginine |
| Tetrahydropentoxyline |
| Theobromine |
| Theophylline |
| Thymol Sulfate |
| Tiglylcarnitine CAR(5:1) |
| Trigonelline |
| Tryptophan |
| Tryptophan betaine |
| Tyramine |
| Tyrosine |
| Uric acid |
| Uridine |
| Urocanic acid |
| Xanthine |
| Xanthosine  **Table S1: Plasma metabolites detected by Liquid Chromatography Mass Spectroscopy** |

| **Demographics** | **n (%) unless stated** | **Range** |
| --- | --- | --- |
| Gender (M) | 20 (64.52%) |  |
| Ethnicity (Caucasian) | 31 (100%) |  |
| Age *(mean [SD]/ years)* | 68.8 [9.5] | 45 - 84 |
| Occupational status (full time/ part time/ retired) | 9 / 2 / 20 |  |
| **Medical History** | | |
| Angina (class I - IV) | 6 (19.3%) |  |
| Dyspnoea (grade 1 to 5) | 28 (90.3%) |  |
| Chronic pulmonary disease | 2 (6.4%) |  |
| Renal Impairment | 19 (61.2%) |  |
| Myocardial infarction | 3 (9.6%) |  |
| Percutaneous intervention | 5 (16.1%) |  |
| Preoperative dysrhythmia | 7 (22.5%) |  |
| Diabetes mellitus | 4 (12.9%) |  |
| Current or ex-smoker | 16 (51.6%) |  |
| Hypertension | 19 (61.2%) |  |
| Hypercholesterolaemia | 18 (58%) |  |
| **Pre-operative assessment** | | |
| LVEF (%) *(median [IQR]) (n=29)* | 55 [8.5] | 20 - 67 |
| Creatinine Clearance (ml/min) *(mean [SD])* | 77 [22.1] | 42 - 124 |
| Logistic EuroSCORE II (%) *(median [IQR])* | 1.6 [1.1] | 0.5 – 9.1 |
| Weight (Kg) *(mean [SD])* | 79.5 [12.6] | 55.4 - 109.8 |
| Height (cm) *(mean [SD])* | 168.9 [7.5] | 156 -189 |
| BMI (Kg/m^2^) *(mean [SD])* | 27.9 [4.4] | 19.1 - 39.3 |
| **Intra-operative** | | |
| Aortic Valve Replacement (AVR) only | 18 (58%) |  |
| AVR + Additional Valve | 1 (3.2%) |  |
| AVR + CABG | 7 (22.5%) |  |
| AVR + other | 5 (16%) |  |
| Bypass Time *(mean [SD])* | 109.4 [30.6] | 61 – 204 |
| Cross-Clamp Time *(median [IQR])* | 83 [30.5] | 61 - 204 |
| **Post-operative outcomes** | | |
| ICU Length of Stay (days; median [IQR]) | 2.1 [1.3] | 1.1-9.9 |
| Hospital Length of Stay (days; median [IQR]) | 6 [3] | 4-13 |
| **Post-operative vasopressors and inotropes** | | |
| Use of Noradrenaline | 23 (74.19%) |  |
| Noradrenaline duration (days; *mean [SD])* | 2 [2] | 1-4 |
| Use of Milrinone | 5 (16.13%) |  |
| Milrinone duration (days; *mean [SD])* | 2 [0] | 2 |

**Table S2: Baseline Characteristics and clinical outcomes**

Data presented as either number (n), frequency (%), mean [SD] or median [IQR] and range. Key: M, male; cm, centimetres; kg, kilograms, μmol/l, micromole per litre; m, metres; ml, millilitres; min, minute; IQR, interquartile range; WHO, World Health Organisation; LVEF, left ventricular ejection fraction; AVR, aortic valve replacement; CABG, coronary artery bypass graft; ICU, intensive care unit.

|  | GDF-15 vs.  Age | GDF-15 vs.  Preop HG | GDF-15 vs.  Pre-op KE | GDF-15 vs.  Pre-op PI | GDF-15 vs.  Pre-op SPPB |
| --- | --- | --- | --- | --- | --- |
| r | 0.530 | -0.445 | -0.347 | 0.398 | -0.423 |
| 95% CI | 0.199 to  0.753 | -0.700 to  0.090 | -0.637 to  0.024 | 0.032 to  0.669 | -0.685 to  -0.062 |
| P Value | 0.003 | 0.014 | 0.059 | 0.030 | 0.020 |
| Number of pairs | 30 | 30 | 30 | 30 | 30 |

**Table S3: Correlation (Spearman analysis) between pre-operative circulating GDF-15 concentration and patient age, pre-operative hand grip (HG), knee extension (KE), muscle quality (rectus femoris pixel intensity: PI) and short physical performance battery (SPPB)**

|  | GDF-15 vs.  FU HG | GDF-15 vs.  FU KE | GDF-15 vs.  FU PI | GDF-15 vs.  FU SPPB |
| --- | --- | --- | --- | --- |
| r | -0.7166 | -0.6781 | 0.7674 | -0.2689 |
| 95% CI | -0.877 to -0.412 | -0.859 to -0.348 | 0.501 to 0.901 | -0.628 to 0.185 |
| P Value | 0.0002 | 0.0005 | <0.0001 | 0.226 |
| Number of pairs | 22 | 22 | 22 | 22 |

**Table S4: Correlation (Spearman analysis) between day 1 circulating GDF-15 concentration and hand grip (HG), knee extension (KE), muscle quality (rectus femoris pixel intensity: PI) and short physical performance battery (SPPB) at follow up (FU)**

| **Metabolites (pre-operative)** | **Robust biweight mid-Correlation (r) with % RFcsa loss at follow-up** | **p value** |
| --- | --- | --- |
| 2-Octenoyl-carnitine | 0.716 | 0.001 |
| N-α-Acetyl-L-arginine | 0.580 | 0.009 |
| 3-Hydroxy-3-methylglutaric acid | -0.578 | 0.010 |
| Sucrose | -0.517 | 0.023 |
| Xanthosine | -0.484 | 0.036 |
| Salicyluric.acid | -0.478 | 0.038 |
| Deoxycholic.acid | 0.468 | 0.043 |
| 4-Hydroxyhippuric acid | -0.464 | 0.045 |
| Salicylic acid | -0.462 | 0.046 |
| Thymol Sulfate | 0.402 | 0.088 |

**Table S5: Top 10 ranked correlation of pre-operative plasma metabolites and proportionate muscle loss at follow up after aortic surgery**

Correlations coefficients between individual metabolites and rectus femoris cross sectional area (RFcsa) change (follow-up/ pre-operative values) were determined using Pearson’s product moment after Robust biweight-mid correlation (n=19).

| **Metabolites (pre-operative)** | **Correlation (r) with % loss of hand grip strength at follow up** | **p value** |
| --- | --- | --- |
| Indoxyl.glucuronide | -0.592 | 0.008 |
| Cotinine | 0.485 | 0.035 |
| Hypoxanthine | -0.473 | 0.041 |
| Xanthine | -0.462 | 0.047 |
| 2,6-Dihydroxybenzoic.acid | 0.455 | 0.050 |
| Dehydroepiandrosterone.Sulfate | -0.450 | 0.053 |
| Glycochenodeoxycholic acid-3-Sulfate | -0.442 | 0.058 |
| N-acetyl-L-carnosine | -0.416 | 0.077 |
| Pregnenolone sulfate | -0.410 | 0.081 |
| Malic acid | -0.391 | 0.098 |

**Table S6: Top 10 ranked correlation of pre-operative plasma metabolites and proportionate hand grip strength at follow up after aortic surgery**

Correlations coefficients between individual metabolites and hand grip strength change (follow-up/ pre-operative values) were determined using Pearson’s product moment after Robust biweight-mid correlation (n=19).

| **Metabolites (day 3)** | **Correlation (r) with % loss of hand grip strength at follow up** | **p value** |
| --- | --- | --- |
| N-Acetylaspartic acid | 0.709 | 0.000 |
| Citric acid | 0.646 | 0.002 |
| Histidine | 0.622 | 0.003 |
| Succinyladenosine | 0.603 | 0.005 |
| Uric acid | 0.574 | 0.008 |
| Pantothenic acid | 0.570 | 0.009 |
| Cis aconitic acid | 0.562 | 0.010 |
| Adipic acid | 0.520 | 0.019 |
| 7-Methylguanine | 0.519 | 0.019 |
| Malic acid | 0.497 | 0.026 |

**Table S7: Top 10 ranked correlation of day 3 plasma metabolites and proportionate hand grip strength at follow up after aortic surgery**

Correlations coefficients between individual metabolites and hand grip strength change (follow-up/ pre-operative values) were determined using Pearson’s product moment after Robust biweight-mid correlation (n=19).

| **Metabolites (pre-operative)** | **Correlation (r) with knee strength pre-op** | **p value** |
| --- | --- | --- |
| Cholic.acid/Ursocholic.acid | 0.633 | 0.004 |
| Hexadecenoylcarnitine | -0.584 | 0.009 |
| Citric.acid | -0.507 | 0.027 |
| 2-Octenoylcarnitine | -0.504 | 0.028 |
| 3-hydroxyisovaleric.acid | 0.480 | 0.037 |
| Phenylalanyltryptophan | -0.464 | 0.046 |
| Cis.aconitic.acid | -0.463 | 0.046 |
| N.acetyl.L.carnosine | 0.456 | 0.050 |
| N.Acetylneuraminic.acid | 0.454 | 0.051 |
| Sucrose | 0.444 | 0.057 |
| **Metabolites (pre-operative)** | **Correlation (r) with knee strength on day 7)** | **p value** |
| 3-hydroxyisovaleric.acid | 0.617 | 0.005 |
| N-Acetylneuraminic.acid | 0.593 | 0.008 |
| Cholic.acid...Ursocholic.acid | 0.582 | 0.009 |
| Citric.acid | -0.566 | 0.012 |
| Cis.aconitic.acid | -0.538 | 0.018 |
| Hexadecenoylcarnitine | -0.536 | 0.018 |
| Pyralline | 0.526 | 0.021 |
| N-acetyl.L-carnosine | 0.499 | 0.030 |
| 2-Octenoylcarnitine | -0.481 | 0.037 |
| Phenylalanine | 0.469 | 0.043 |
| **Metabolites (pre-operative)** | **Correlation (r) with knee strength at follow up** | **p value** |
| 3.hydroxyisovaleric.acid | 0.691 | 0.001 |
| N-Acetylneuraminic.acid | 0.564 | 0.012 |
| Phenylalanine | 0.544 | 0.016 |
| Dehydroepiandrosterone.Sulfate | 0.538 | 0.018 |
| N-acetyl.L.carnosine | 0.536 | 0.018 |
| Leucine | 0.517 | 0.024 |
| Propionylcarnitine | 0.488 | 0.034 |
| Pyralline | 0.486 | 0.035 |
| Leucylleucine | 0.478 | 0.039 |
| Pyroglutamic.acid | 0.476 | 0.040 |

**Table S8: Top 10 ranked correlation of pre-operative plasma metabolites and knee extension measured pre-operatively, at day 7 and at follow-up**

Correlations coefficients between individual metabolites and knee extension strength were determined using Pearson’s product moment after Robust biweight-mid correlation (n=19).
