# Supplementary figures and images for: Mitochondrial Dysfunction Contributes to Sustained Muscle Loss After Cardiac Surgery: A Prospective Observational Study
Source: J Cachexia Sarcopenia Muscle. 2025 Aug 21;16(4):e70051. doi: 10.1002/jcsm.70051 (PMC12370447; doi:10.1002/jcsm.70051)

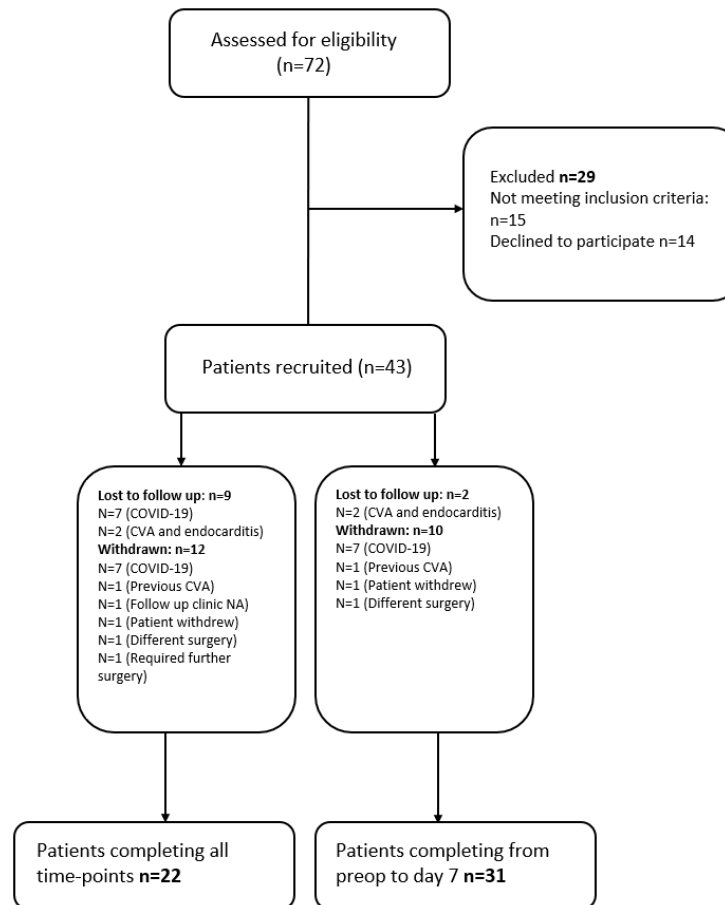

Figure S1

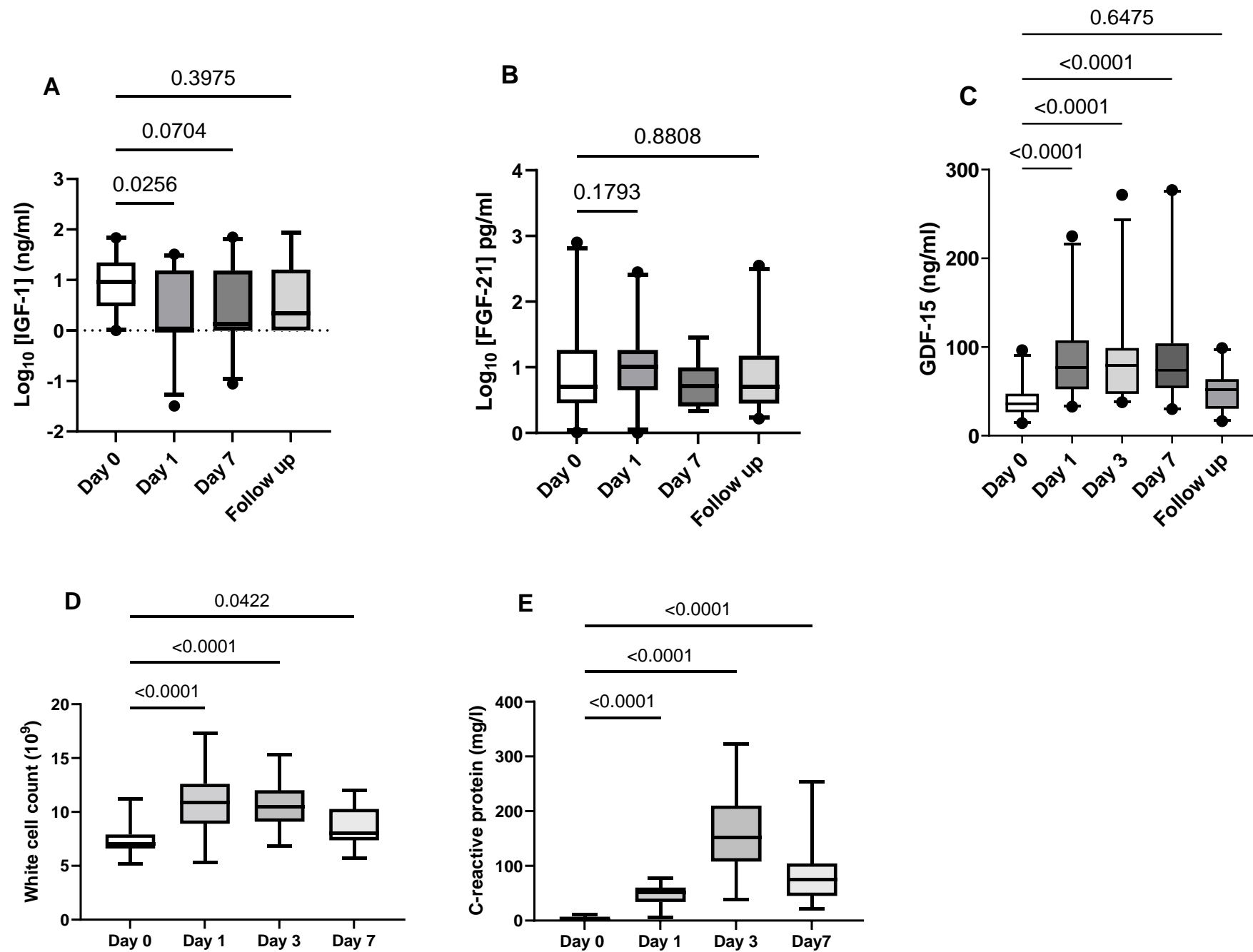

Figure S2

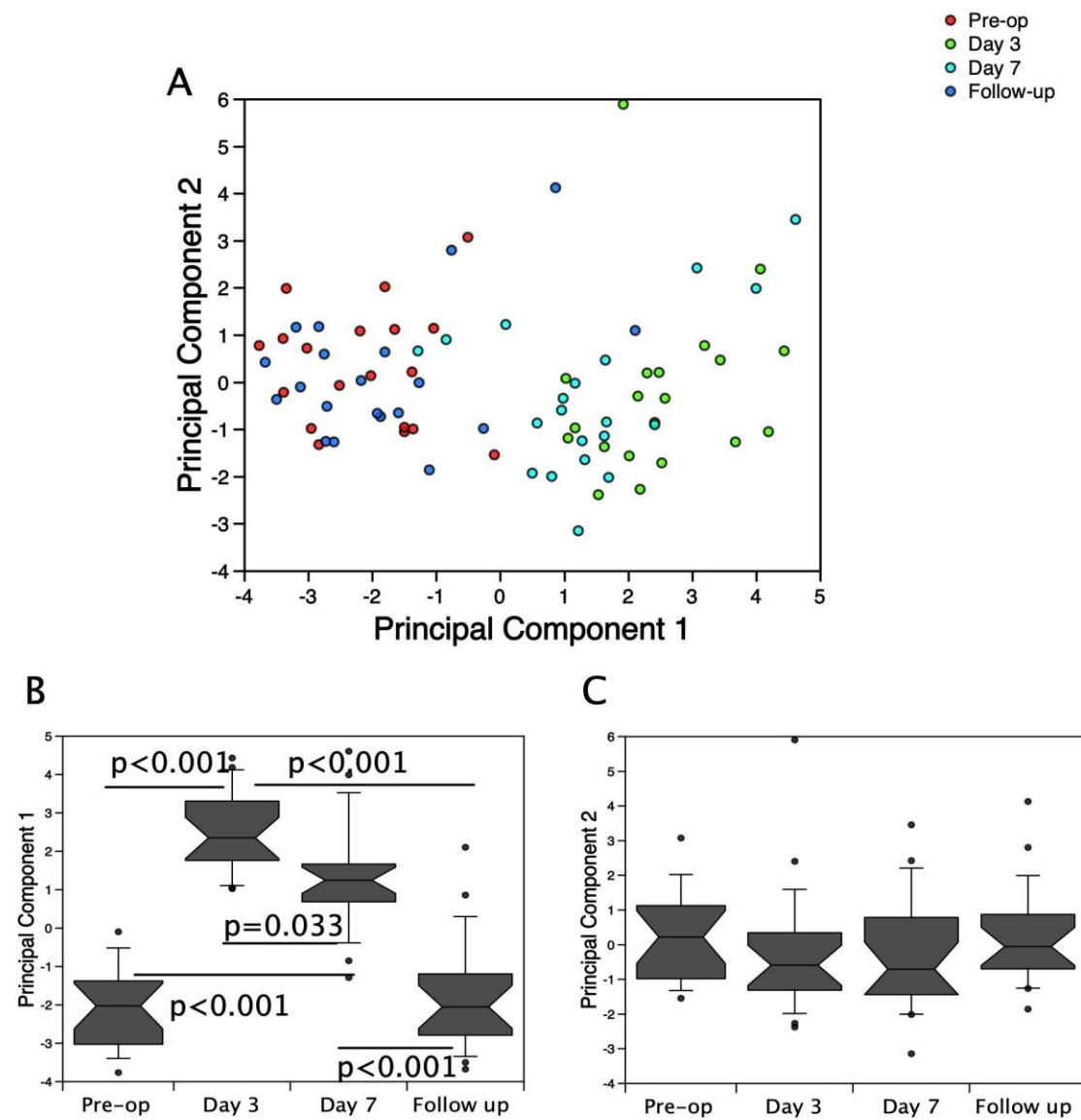

Figure S3

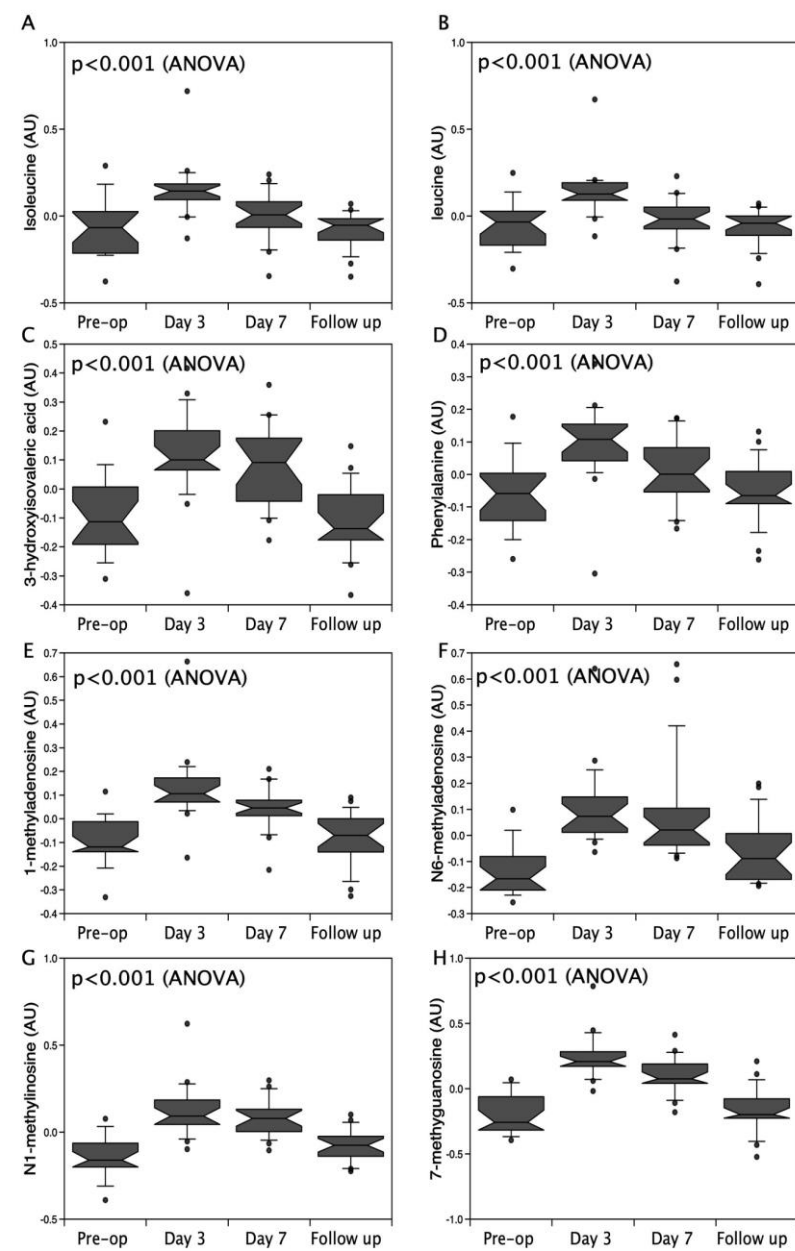

Figure S4

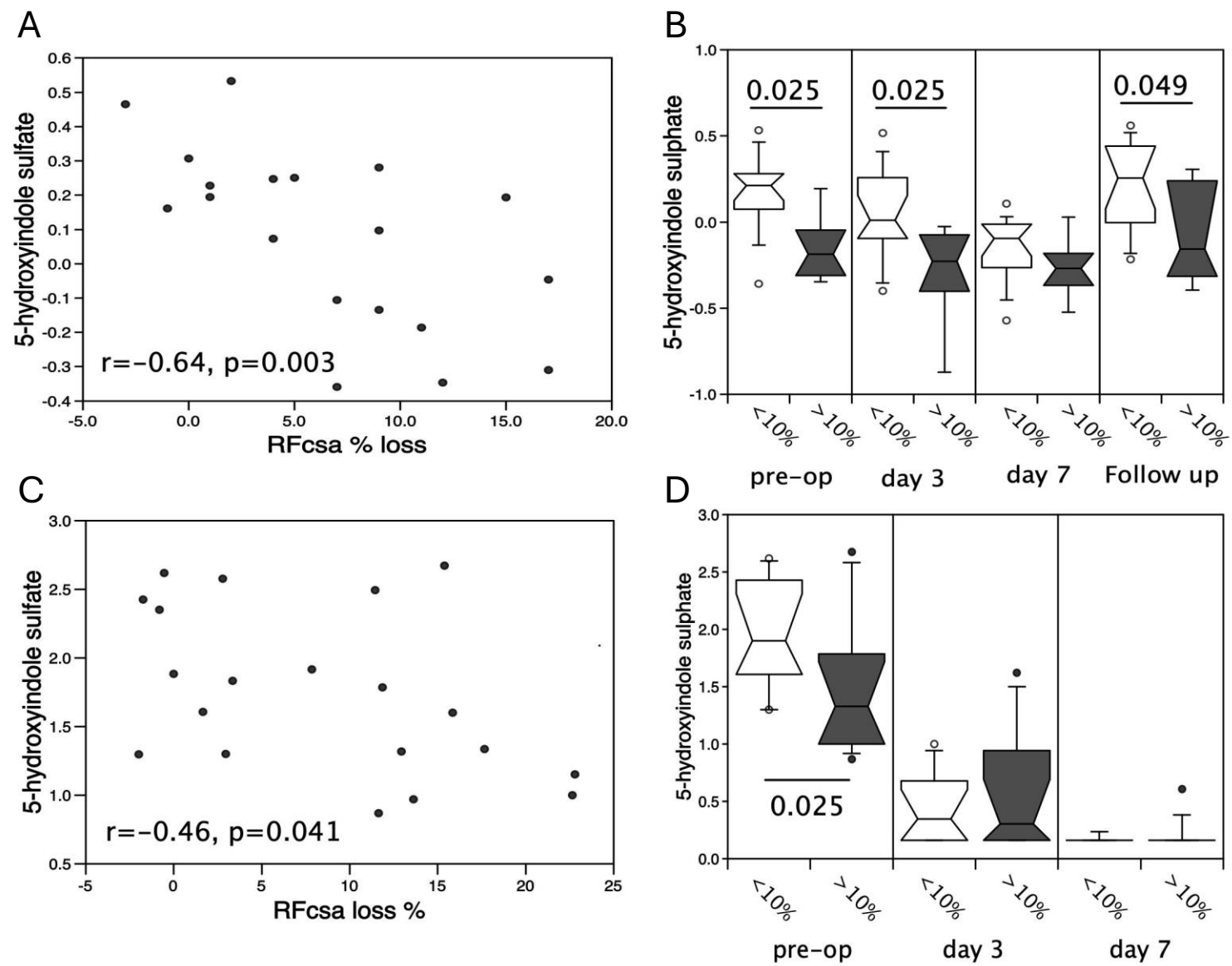

Figure S5

Supplement: Supplementary file 3 — Figure S1: Modified CONSORT diagram. CVA, cerebral vascular accident; DNA, did not attend. Figure S2: Time course after aortic surgery of circulating mediators of muscle homeostasis and markers of inflammation. A, B, C: Plasma levels of insulin‐like growth factor (IGF)‐1, fibroblast growth factor (FGF)‐21 and growth and differentiation factor (GDF)‐15 were quantified by ELISA. D: The white cell count was derived from Routine full blood count Coulter counter data. E: C‐reactive protein measured in the clinical laboratory. Data analysed using Dunn's multiple comparisons against pre‐op baseline (Day 0: median [IQR, 95% CI], n = 22–30). Figure S3: The effect of aortic surgery on patients' plasma metabolites. A: Principal component analysis was carried out using all available metabolite data following normalisation. Scatter plot of PC1 and PC2 for all samples shows marked change along PC1 from Days 1 to 3 with a small return towards pre‐op values on Day 7. Follow‐up is indistinguishable from Day 0. B: Median PC1 values for each day were plotted with interquartile range and outliers at 90% shown as individual points. There was a marked increase in PC1 between Days 0 and 3 that returned to baseline at follow‐up. C: Median PC2 values for each day are plotted with interquartile range and outliers at 90% shown as individual points. PC2 did not vary significantly over the time course, comparisons by two‐way ANOVA comparing the effect of time on plasma metabolites throughout, n = 19. Figure S4: Time course after aortic surgery of circulating amino acids (A to D) and nucleotide metabolites (E to H) from patients' plasma. Plasma amino acids and some of their metabolites (A–D: isoleucine, leucine, phenylalanine and 3‐hydroxyisovaleric acid) and nucleotide metabolites (E–H: N6‐methyl adenosine, N1‐methyl inosine and 7‐methyl‐guanosine) showed that these were all elevated on Day 3 compared with presurgery and returned to baseline by follow‐up (median with interquartile range and out [file JCSM-16-e70051-s003.pdf]
